# Development and validation of a meta-learner for combining statistical and machine learning prediction models in individuals with depression

**DOI:** 10.1186/s12888-022-03986-0

**Published:** 2022-05-16

**Authors:** Qiang Liu, Georgia Salanti, Franco De Crescenzo, Edoardo Giuseppe Ostinelli, Zhenpeng Li, Anneka Tomlinson, Andrea Cipriani, Orestis Efthimiou

**Affiliations:** 1 Department of Psychiatry, Warneford Hospital, University of Oxford, Oxford, OX3 7JX UK; 2 Oxford Precision Psychiatry Lab, NIHR Oxford Health Biomedical Research Centre, Oxford, UK; 3 Institute of Social and Preventive Medicine, University of Bern, Bern, Switzerland; 4 Oxford Health NHS Foundation Trust, Warneford Hospital, Oxford, UK; 5 Institute of Primary Health Care (BIHAM), University of Bern, Bern, Switzerland

**Keywords:** Depression, PHQ-9, Dropout, Machine learning, Statistical model

## Abstract

**Background:**

The debate of whether machine learning models offer advantages over standard statistical methods when making predictions is ongoing. We discuss the use of a meta-learner model combining both approaches as an alternative.

**Methods:**

To illustrate the development of a meta-learner, we used a dataset of 187,757 people with depression. Using 31 variables, we aimed to predict two outcomes measured 60 days after initiation of antidepressant treatment: severity of depressive symptoms (continuous) and all-cause dropouts (binary). We fitted a ridge regression and a multi-layer perceptron (MLP) deep neural network as two separate prediction models (“base-learners”). We then developed two “meta-learners”, combining predictions from the two base-learners. To compare the performance across the different methods, we calculated mean absolute error (MAE, for continuous outcome) and the area under the receiver operating characteristic curve (AUC, for binary outcome) using bootstrapping.

**Results:**

Compared to the best performing base-learner (MLP base-learner, MAE at 4.63, AUC at 0.59), the best performing meta-learner showed a 2.49% decrease in MAE at 4.52 for the continuous outcome and a 6.47% increase in AUC at 0.60 for the binary outcome.

**Conclusions:**

A meta-learner approach may effectively combine multiple prediction models. Choosing between statistical and machine learning models may not be necessary in practice.

**Supplementary Information:**

The online version contains supplementary material available at 10.1186/s12888-022-03986-0.

## Background

Since routinely collected clinical, imaging, and multi-omics information have entered the big data era [1], more and more analyses in medical research are undertaken using machine learning models [2–5]. The debate between machine learning and statistical models is, however, ongoing. Several articles have compared these approaches and provided an assessment of their relative performance [6–13]. Generally, machine learning models are expected to perform better when there are many potentially relevant predictors, and especially when there are non-linear relationships between predictors and the outcomes as well as interactions between predictors. However, they may be more sensitive to initial, randomly allocated parameters, and noise in the training dataset than conventional statistical models, i.e., they may be more prone to overfitting [14]. Such issues may put constraints on their generalizability [15]. Another commonly criticized issue is their lack of transparency, as they sacrifice interpretability for predictive power [16]. Furthermore, when it comes to clinical predictions, particularly in low-dimensional settings with large datasets consisting of mostly linearly separable predictors, there is no evidence of superior performance of machine learning over statistical models [8, 17, 18]. However, this has been disputed by other researchers, who claimed that such results were due to the specific choices of datasets and models [19, 20]. In any case, an arguably good strategy is to deploy machine learning models only when there are suspected non-linear relationships and interactions. In practice, of course, it is not easy to decide a priori whether this is the case and whether machine learning methods may offer any benefit over simpler approaches.

Here we suggest that choosing between the two methods may not be necessary. Instead, a combined approach, i.e., a “meta-learner”, can be employed to take the best out of its world. In this paper, we used a dataset from patients with depression to illustrate the development of two meta-learners using a machine learning and a statistical model as base-learners. In what follows, we first present the dataset and then describe in detail the development of the meta-learners.

## Methods

Table 1 provides a glossary of all terms used in the paper. Below we describe methods in more detail.

**Table 1:** Glossary

Term	Definition
Area under the receiver operating characteristic curve (AUC)	A discrimination metric for classification problems, measuring the area under the entire receiver operating characteristic curve. AUC ranges from 0 to 1 with higher values indicating better performance.
Base-learner	A single, stand-alone statistical or machine learning model built for predicting a continuous or a binary outcome.
Bootstrapping	Random sampling data with replacement.
Calibration-in-the-large	A method for measuring the agreement between observed outcomes and predictions for classification problems, where the average predicted probability is compared with the observed event rate. A mismatch indicates that the model over- or underestimates the risk on average.
Deep neural network	A type of machine learning model that resembles how neurons in human brain work.
Mean absolute error (MAE)	MAE measures the average magnitude of errors, i.e., the difference between true/observed values and their predictions. Lower MAE indicates better performance.
Meta-learner	A statistical or machine learning model that uses as input the output of other models (i.e., base-learners), to predict an outcome of interest.
Multi-layer perceptron (MLP)	The simplest deep neural network model with multiple stacked hidden layers.
Overfitting	The case when a model fits too closely to the data used to develop the model (training data), but performs badly on new, testing data.
Permutation feature importance	A method to evaluate the importance of predictors used in machine learning models, by measuring the decrease in model performance when the predictor’s values are randomly shuffled.
Ridge regression	A statistical regression model which uses a penalized likelihood. The penalty has the effect of shrinking the estimated coefficients so that the model does not yield extreme predictions.

### Study design and patients

We aimed to predict two outcomes: severity of depressive symptoms (continuous outcome) and all-cause dropout rate (binary outcome) measured 60 days after the initiation of antidepressant treatment. We used data from QResearch, a primary care research database in the UK (https://www.qresearch.org/). From patients registered in QResearch since 1st Jan 1998, we included patients aged > 18 years, who were diagnosed with depression and prescribed fluoxetine [21]. We excluded patients with a previous episode of depression or a previous prescription of antidepressants in the year before. We identified 187,757 patients for the all-cause dropout analysis, 16,384 of whom were used for the severity of depressive symptoms analysis. Detailed inclusion and exclusion criteria can be found in Additional file 1.

All-cause dropout was defined as discontinuation of fluoxetine within 60 days due to any reason. Patients were assumed to have discontinued treatment if they 1) had a gap of more than 30 days between the end of the previous prescription and the start of the next prescription, 2) switched to another antidepressant, or 3) were prescribed an additional antidepressant, a mood stabilizer, or an antipsychotic (augmentation). The severity of depressive symptoms was measured with Patient Health Questionnaire-9 (PHQ-9), which ranges from 0 to 27 [22]. If a patient did not have outcome data reported 60 days after the diagnosis of depression, we considered a valid measurement of any outcome recorded between 21 and 90 days. We transformed other depression rating scales into PHQ-9 scores using validated approaches [23, 24]. A description of the clinical and demographic characteristics of patients is shown in Additional file 1: Tables S1 and S2.

We selected candidate predictors for the models based on a review of the current literature [25]. We considered baseline characteristics such as demographic variables, condition-specific variables (e.g., depression severity), information relevant to previous treatments and comorbidities. A detailed list of predictors and a mock dataset of five patients are presented in Additional file 1: Table S3.

### Base-learners to generate predictions

**Fig. 1:**
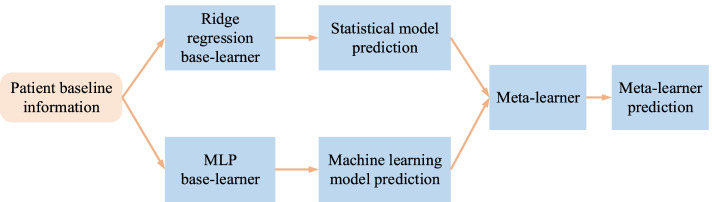
Meta-learner architecture. Patient-level baseline information was used to independently develop the base-learners (i.e., a ridge regression model and an MLP). Their predictions were in turn used as sole predictors of the meta-learners. We explored two different types of meta-learners, namely linear regression (logistic regression for the binary outcome), and MLP. MLP: multi-layer perceptron

We used two base-learners to develop the meta-learners (Fig. 1. Meta-learner architecture.), although more models could be used in practice. Patient baseline information and observed outcomes were used to train the base-learners. Predictions from the base-learners were in turn used as sole predictors in meta-learners. The statistical base-learner was a ridge regression with restricted cubic splines (four knots) for the continuous predictors (i.e., age, BMI, baseline depression severity). The machine learning base-learner was a multi-layer perceptron (MLP) deep neural network (3 hidden layers, 256 neurons per layer).

To prepare the training set (base-learner predictions) for a meta-learner and, at the same time, prevent overfitting when training a meta-learner, base-learner predictions should be made on out-of-sample data. One approach is to randomly split the original dataset into two sets and develop base-learners using one. Next, use the base-learners to make predictions on the other set and train the meta-learner on this set only. This simple approach is however wasteful and can be problematic for smaller datasets. To fully leverage data, resampling is commonly used to generate out-of-sample predictions for all patients in the dataset [26, 27]. In this paper, we used bootstrapping [28]. Specifically, we created a bootstrap sample where we separately fitted the base-learners and made predictions on out-of-sample patients, i.e., patients included in the original dataset but not included in this bootstrap sample. We repeated the procedure many times, obtaining multiple predictions for each patient and from each base-learner. We averaged these predictions so that for each patient we obtained a single out-of-sample prediction from each base-learner. These predictions were then used to train the meta-learners.

### Developing meta-learners

Meta-learners belong to the family of so-called ensemble learning methods. In ensemble learning, multiple base-learners are combined in a sequential or parallel manner to develop a new model. A meta-learner is expected to yield better predictive performance than each of its parts [29]. Various combination techniques, such as majority voting and weighted averaging, can be deployed for combining predictions from base-learners. Meta-learners that use base-learners of more diverse structures are generally expected to perform better [27, 30, 31].

Here we focused on one of the ensemble learning techniques, i.e., stacked generalization, to construct meta-learners. In this way, meta-learners can be any statistical or machine learning model. They take as input predictions generated by base-learners and output a single prediction (Fig. 1. Meta-learner architecture.).

We explored two meta-learners. The first meta-learner was a simple linear (or logistic, for the binary outcome) regression model, where we used the out-of-sample predictions obtained from the base-learners as the only two predictors to predict outcomes. The second meta-learner was an MLP (3 hidden layers, 256 neurons per layer), where the same predictors and observed outcomes were used for training. The implementation of the MLP is detailed in Additional file 1: Table S5.

### Model evaluation metrics and measuring the importance of predictors

To evaluate model performance for the base- and meta-learners, we used mean absolute error (MAE) for PHQ-9 prediction, and the area under the receiver operating characteristic curve (AUC) and calibration-in-the-large for dropout prediction. Readers should note that percent changes of AUC reported in the paper were calculated after subtracting the baseline of AUC (0.5) from all estimates. Implementation details are given below.

In statistical models, the importance of each predictor can be assessed by the change in a metric, when this predictor is omitted from the model. An alternative approach to estimate the importance of a predictor is the permutation feature importance method [32, 33], which measures the decrease in a model’s performance when a predictor in the testing data is randomly shuffled. The model is fitted on the training data as usual, and predictions are generated from the testing data. Since values of this predictor are randomly shuffled in the testing data, the model generally yields worse predictions, e.g., increased MAE or decreased AUC. More substantial changes to these metrics indicate a more predictive covariate. Here, in addition to evaluating the importance of each predictor for the MLP base-learner, we used this method to estimate the importance of each base-learner for the MLP meta-learner.

### Multiple imputations for missing data

We did not exclude subjects with missing predictors as this would greatly reduce the study sample size, precision and power [34]. We deployed a multiple imputation method [35] with additive regressions to impute missing values. We followed previous recommendations [34, 36–39] and included outcome data for imputation. For continuous predictors, we used restricted cubic splines to match the statistical analysis method. We generated 10 imputed datasets. Regarding the type of missingness, we compared the observed outcomes for patients with missing covariates to those with observed covariates. Results are shown in Tables S6 and S7 in Additional file 1. Although the missing at random assumption is generally untestable [40], the multiple imputation approach potentially offers some protection from bias [41]. We did not use the imputed PHQ-9 outcomes for model development or evaluation.

### Evaluation of model performance

We generated 20 bootstrap samples from each of the imputed datasets (200 samples in total) and fitted ridge regression (with splines as indicated above) and MLP base-learners using all 31 predictors.

In more detail, for each imputed dataset we created a random bootstrap sample. The base-learners were developed in the bootstrap sample (i.e., this was the training set). We used out-of-sample patients, i.e., patients not included in the bootstrap sample, to make predictions using the developed models. By construction, a bootstrap sample has the same size as the original dataset and contains on average approximately 70% of the patients found in the original dataset, leaving approximately 30% of the patients for testing. We calculated measures of performance (MAE, AUC, calibration) for each base-learner. We repeated this bootstrap procedure 20 times per imputed dataset. In the end, we summarized measures of performance for the base-learners, after averaging the 200 sets of values. At this point, for each patient, we had obtained multiple, out-of-sample predictions from each of the base-learners. Next, we averaged these predictions from each base-learner at the patient level. These two predictions per patient (i.e., one per base learner) were used as inputs to develop the two meta-learners.

To evaluate the uncertainty around the estimated measures of performance of the meta-learners, we performed an additional bootstrap analysis (details are given in Additional file 1). Finally, we used permutation feature importance to assess the importance of predictors. For each base- and meta-learner, we randomly selected 10 models already trained on their corresponding bootstrap samples as described above, and shuffled each predictor of their associated out-of-sample data 100 times.

As a sensitivity analysis, we evaluated different data pre-processing techniques, namely data standardization and data normalization, and compared them to using non-pre-processed data, through the ridge regression base-learner for PHQ-9 score prediction.

### Internal-external validation

To evaluate the generalizability of our model’s performance and to ensure the transportability of their results in different (but similar) settings, we further compared the base- and meta-learners using a so-called internal-external cross-validation method [42, 43]. More specifically, our dataset was collected in 10 different regions of England. We took one region out of the data at a time and used the remaining 9 regions to develop all base- and meta-learners. We then tested all models on the left-out region. Next, we cycled through all regions, leaving a different region out each time. In the end, we reported the measures of performance by region as well as the overall average. Details are given in Additional file 1.

### Implementation details

All analyses were carried out on a desktop computer with an Intel Xeon Gold 6246 12 cores CPU and an Nvidia Tesla V100 32G GPU. Data cleaning was implemented in Stata [44]. Imputation and ridge regression base-learner fitting were carried out using Hmisc in R [45]. MLP base-learner training, meta-learner training, model evaluation and feature importance calculation were conducted in TensorFlow and Python [46, 47].

We provide additional details on how to use the meta-learner to predict for a new patient in Additional file 1. We provide online code for developing a meta-learner in Python: https://github.com/oceanlq/Meta-learner.

## Results

Overall, in our illustrative example, we found that the meta-learners led to better predictive performance as compared to any of the base-learners, for both the continuous and the binary outcome. Below we present results in detail.

### Severity of depressive symptoms (PHQ-9 score)

**Fig. 2:**
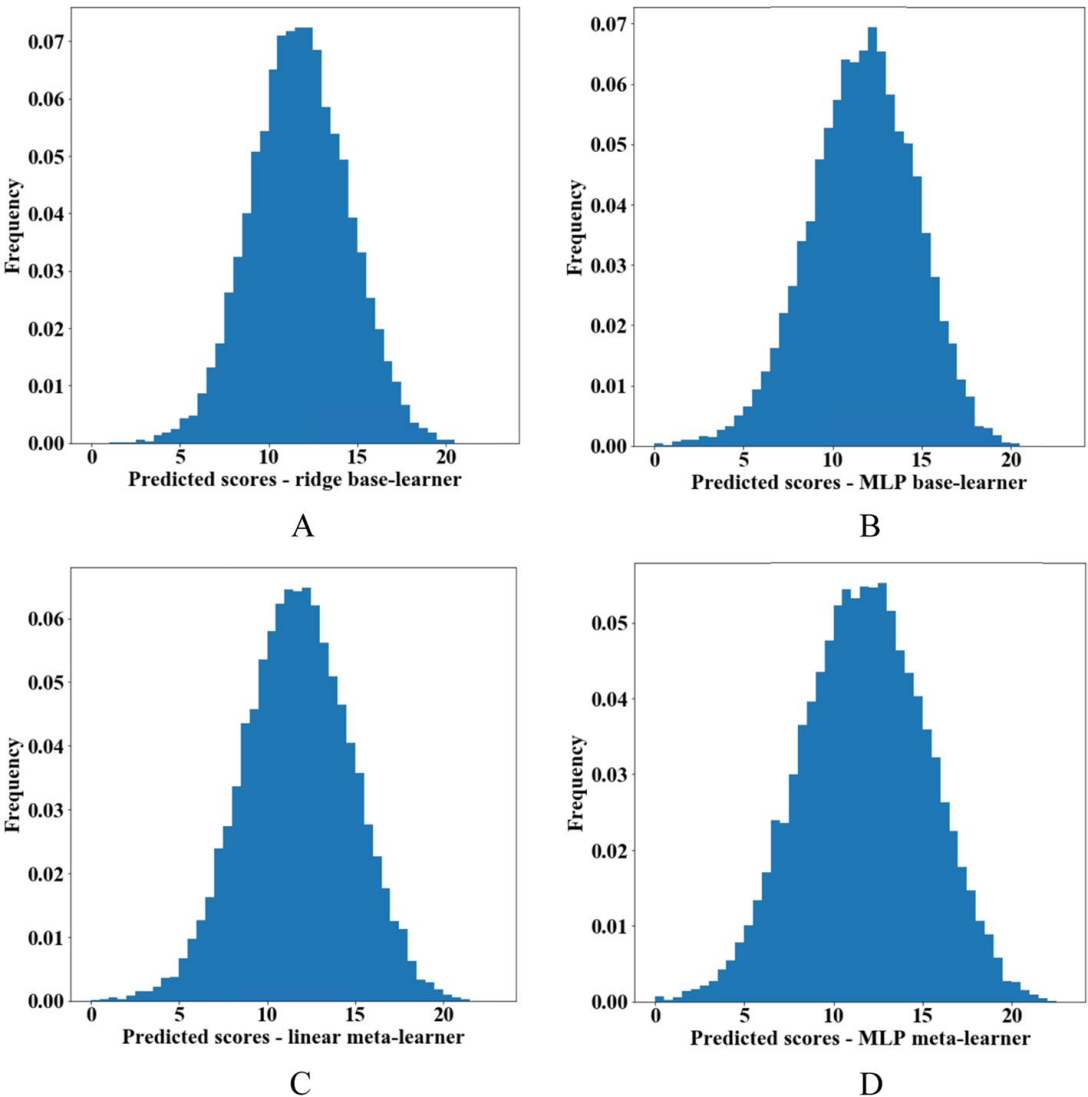
Histograms of predicted Patient Health Questionnaire-9 (PHQ-9) scores. **A** Ridge regression base-learner. **B** Multi-layer perceptron (MLP) base-learner. **C** Linear regression meta-learner without regularization. **D** MLP meta-learner

We provide histograms of the predicted PHQ-9 scores from each base- and meta-learner in Fig. 2. Generally, we observed a modest predictive performance for all models and predictions of the meta-learners were very similar to each other. We also provide pairwise scatterplots to compare predictions among the various models, as well as between predicted and observed outcomes (Additional file 1: Fig. S1).

We show estimates of all performance measures with 95% CIs in Table 2 and Additional file 1: Fig. S2. Both meta-learners had lower MAEs compared to the base-learners. The MAEs of predicted PHQ-9 score for the base-learners were almost identical, around 4.63 on the PHQ-9 scale. Compared with the best performing base-learner (MLP), the decrease of MAE was 2.11% for the linear regression meta-learner, at 4.54 in PHQ-9, and 2.49% for the MLP meta-learner at 4.52. Results from the internal-external cross-validation are reported in Additional file 1: Table S8. The base-learner performed similarly to what they had performed in the whole dataset. Again, we saw that both meta-learners had lower MAEs compared to the base-learners, and that, as expected, they performed slightly worse than the meta-learners in the main analysis described above. Compared with the best performing base-learner (MLP), the decrease of MAE was 1.86% for the linear regression meta-learner, at 4.55 in PHQ-9, and 2.27% for the MLP meta-learner at 4.53. The heterogeneity of model performance was relatively small, with the MLP meta-learner performing best in the Yorkshire and Humber region, with the MAE at 4.23, and worst in the North East England region, with the MAE at 4.73.

**Table 2:** Evaluation on post treatment Patient Health Questionnaire (PHQ-9) and all-cause dropout predictions made by the statistical, machine learning base-learners and meta-learners

	PHQ-9 score MAE^*^ [95% CI^†^]	Dropout AUC^‡^ [95% CI]
Base-learner ridge regression	4.64 [4.57, 4.71]	0.597 [0.594, 0.601]
Base-learner MLP^§^	4.63 [4.57, 4.71]	0.598 [0.594, 0.601]
Meta-learner linear/logistic regression	4.54 [4.48, 4.60]	0.604 [0.600, 0.606]
Meta-learner MLP	4.52 [4.45, 4.59]	0.604 [0.600, 0.606]

^*^Mean absolute error.

^†^Confidence interval, calculated as the 2.5th to the 97.5th percentile of bootstrap estimates.

^‡^Area under the receiver operating characteristic curve.

^§^Multi-layer perceptron.

The permutation feature importance analysis of the patient predictors used in the two base-learners led to similar conclusions, identifying PHQ-9 baseline score to be the dominant predictor, with an increase of around 20% in MAE, 25 times larger than the second most important predictor (Townsend deprivation index). Moreover, demographic variables were generally found to be more predictive than variables related to comorbidities. Detailed results of all analyses are provided in Additional file 1: Figs. S5, S7, S9, S11 and Tables S10, S12. We also performed a permutation feature importance analysis on the two meta-learners, to assess the relative importance of base-learner predictions, as shown in Table 3. Conclusions were similar for both ridge and MLP meta-learners, showing that the statistical base-learner contributed much more than the machine learning one (around 80 times more for the ridge meta-learner, 14 times more for the MLP meta-learner), indicating that although the base-learners performed similarly, the meta-learners mainly used the ridge base-learner predictions, and only partly the prediction from the MLP base-learner, for minor correction and fine-tuning non-linear patterns.

**Table 3:** Permutation feature importance values of the meta-learners

Outcome	Meta-learner	Base-learner	Change of measures (%)
PHQ-9,^**^ measured by MAE^††^	Linear regression	Ridge regression	24.78
		MLP^‡‡^	0.30
	MLP	Ridge regression	31.70
		MLP	2.34
Dropout, measured by AUC^§§^	Logistic regression	Ridge regression	0.83
		MLP	5.21
	MLP	Ridge regression	0.42
		MLP	2.67

^**^Patient Health Questionnaire-9.

^††^Mean absolute error.

^‡‡^Multi-layer perceptron.

^§§^Area under the receiver operating characteristic curve.

Results from the sensitivity analysis that used different data pre-processing techniques are presented in Additional file 1: Table S14. All results were very similar to the main analysis.

### All-cause dropouts

**Fig. 3:**
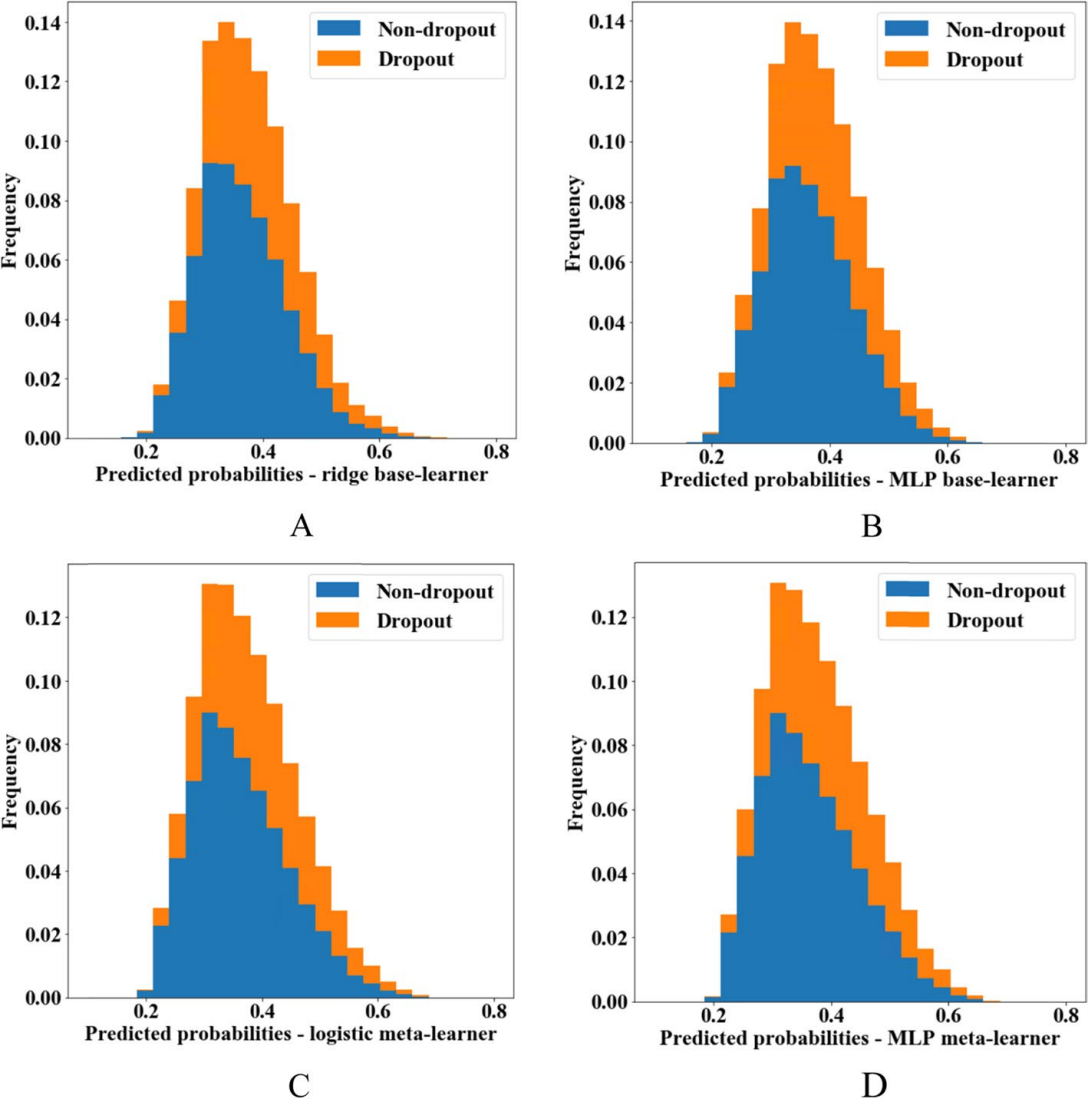
Histograms of predicted probabilities of dropout. Colors are according to observed outcomes. **A** Ridge regression base-learner. **B** Multi-layer perceptron (MLP) base-learner. **C** Logistic regression meta-learner without regularization. **D** MLP meta-learner

We provide histograms of the predicted probabilities of dropout from all models in Fig. 3, and pairwise scatterplots to visualize their agreement in Additional file 1: Fig. S3. From the plots, it is obvious that all models had a low discrimination ability. Predictions from the meta-learners were very similar to each other, although predictions from the two base-learners were not always in good agreement.

Next, we found that the AUCs of the base-learners were very similar to each other as well. Both meta-learners had slightly higher AUCs compared to the base-learners (Table 2 and Additional file 1: Fig. S4). The increase of AUC was around 6.50% for both meta-learners, with the MLP meta-learner performing slightly better than the logistic regression meta-learner. The observed dropout ratio in our dataset was 37.50%. We examined calibration-in-the-large by comparing this value with the average probability of an event estimated from our models. We found that all models gave values very close to the observed, within 37 to 38%. We report results from the internal-external cross-validation in Additional file 1: Table S9. Again, we saw that both meta-learners had slightly higher AUCs compared to the base-learners, but slightly worse than the meta-learners in the main analysis. Compared with the best performing base-learner (MLP), the increase of AUC was around 5.10% for both meta-learners. Finally, we found small evidence of variability in model performance across different regions, with the MLP meta-learner performing best in the North West England region, with the AUC at 0.61, and worst in the Yorkshire & Humber region, with the AUC at 0.59.

The permutation feature importance for the predictors of the two base-learners gave similar results. Ethnicity was the most predictive variable, with an AUC decrease of 3 and 2% for ridge and MLP base-learners respectively, followed by Townsend deprivation with a decrease of around 2%. The decrease was below 0.5% for all other predictors. Detailed results are shown in Additional file 1: Figs. S6, S8, S10, S12 and Tables S11, S13. The permutation feature importance evaluation on the two meta-learners is shown in Table 3. In contrast to the PHQ-9 prediction, we found that for this outcome, for both meta-learners the machine learning base-learner contributed around 6 times more than the statistical model one.

## Discussion

A prevalent view among practitioners is that statistical methods are more suitable when the number of predictors is relatively small, while machine learning models are more useful for bigger datasets and when non-linear relationships among the predictors and the outcome are suspected [48]. Accordingly, in a recent paper, Austin et al. [17] showed in simulations that in the “large N, small p” setting, statistical learning methods perform well, suggesting that more complicated methods may bring small benefits. Recently, however, there has been a call for an integration of the two approaches, so that they can complement rather than antagonize each other [49]. This paper took a step in this direction. We presented how meta-learners based on ensemble learning can effectively synthesize statistical and machine learning models for predicting continuous or binary outcomes, and potentially improve performance. Thus, our results suggest that choosing between statistical and machine learning models with respect to predictive tasks in the medical domain may not always be necessary.

More specifically, we showed that a meta-learner, even the simplest one, can potentially give better predictive performance than its constituent parts, i.e., the base-learners. As an illustrative example, we used a dataset from patients with depression, where we aimed to predict depression severity and probability of dropping out from antidepressant treatment due to any reason at 60 days. Both outcomes are generally relevant for clinical decision making, i.e., for deciding upon treatment initiation.

In our example, we found that the MLP meta-learner performed slightly better than the linear (logistic) regression meta-learner, for both outcomes in both cases, indicating the MLP meta-learner picked up some nonlinearity from the base-learner predictions. Thus, for better predictive performance, a more advanced meta-learner might be preferable in practice [50]. We also conducted further analyses, where we reused patient-level covariates as additional predictors for the meta-learners (Additional file 1: Table S15). We found that incorporating the patient covariates as additional meta-learner input may potentially further benefit the performance of a meta-learner.

One general limitation of the meta-learners we presented is the increased complexity in terms of model building, which requires expertise in different domains. Moreover, although in our example we saw some increase in performance when using the meta-learners, this gain was clinically insignificant. One possible explanation is that in this dataset the outcomes were mostly linearly dependent on the predictors, so that both statistical and machine learning base-learners utilized the full potential of the dataset, thus not leaving much room for the meta-learners to improve performance. Another reason for the modest gain in performance might be that in terms of stacked generalization, the biggest gains are usually observed when base-learners have high variability, i.e., when they are trapped in different local minimums and generate uncorrelated, diverse predicted values [27]. In our example, however, both base-learners seem to have reached a global minimum, generated optimal performance on their own and predicted broadly similar predictions. One important limitation of this study was that one predictor, namely the PHQ-9 baseline score, had 75% missing values. In this case, multiple imputations might introduce bias and possibly a complete case analysis might have been preferable. We decided, however, not to exclude this predictor from our analysis because it was a priori deemed to be one of the most important patient baseline characteristics. Moreover, excluding patients with missing PHQ-9 baseline scores would considerably reduce our sample size.

Finally, readers should note that in this paper we did not predict relative effects between treatments. This would require having in the dataset patients on different treatments, the inclusion of treatment itself as a predictor in the model, and the use of causal inference methods. Ideally, randomized data should be used for such an aim – although observational data could also be used thanks to methodological advancements in recent years. However, in this paper we did not consider relative treatment effects at all, and we only focused on the case of predicting an absolute outcome.

## Conclusion

The proposed meta-learner has the potential to efficiently combine statistical and machine learning models for joint prediction. With appropriate adjustment, the meta-learner may be employed as a handy alternative solution to predictive tasks in the medical domain. As practical advice, we corroborate previous recommendations [51] by suggesting the use of classical statistical methods for most clinical research questions, which typically do not involve large datasets or many potential predictors. Following Occam’s razor, we especially recommend classical methods when there is a prior indication that predictors mainly affect the outcome in an additive manner and when interactions between them can either be prespecified or play a relatively smaller role. Conversely, for larger datasets, and especially when non-additive effects are suspected, researchers can explore both statistical methods and machine learning approaches and follow a meta-learner approach to combine, rather than compare, the two methods. An interesting area of future research is to further explore the performance of meta-learners in simulated datasets and assess their performance on additional real examples.

## Supplementary Information

**Additional file 1.**

## Data Availability

The data that support the findings of this study are available by application through QResearch (https://www.qresearch.org/, email: qresearch@phc.ox.ac.uk). Owing to the sensitivity of clinical information, access is dependent on receiving research approval from the QResearch Scientific Committee. The codes are available on request from the corresponding author – Qiang Liu. A sample code for developing a meta-learner in Python can be found on GitHub (https://github.com/oceanlq/Meta-learner).

## Abbreviations

AUC: Area under the receiver operating characteristic curve
MAE: Mean absolute error
MLP: Multi-layer perceptron
PHQ-9: Patient Health Questionnaire-9

## References

[1] Iniesta R, Stahl D, McGuffin P (2016). Machine learning, statistical learning and the future of biological research in psychiatry. Psychol Med.

[2] Chen M, Hao Y, Hwang K, Wang L, Wang L (2017). Disease prediction by machine learning over big data from healthcare communities. IEEE Access.

[3] Christiansen EM, Yang SJ, Ando DM, Javaherian A, Skibinski G, Lipnick S, Mount E, O’Neil A, Shah K, Lee AK (2018). In silico labeling: predicting fluorescent labels in unlabeled images. Cell.

[4] Senior AW, Evans R, Jumper J, Kirkpatrick J, Sifre L, Green T, Qin C, Žídek A, Nelson AW, Bridgland A (2020). Improved protein structure prediction using potentials from deep learning. Nature.

[5] Liu Q, Vaci N, Koychev I, Kormilitzin A, Li Z, Cipriani A, Nevado-Holgado A (2022). Personalised treatment for cognitive impairment in dementia: development and validation of an artificial intelligence model. BMC Med.

[6] Singal AG, Mukherjee A, Elmunzer BJ, Higgins PD, Lok AS, Zhu J, Marrero JA, Waljee AK (2013). Machine learning algorithms outperform conventional regression models in predicting development of hepatocellular carcinoma. Am J Gastroenterol.

[7] Westreich D, Lessler J, Funk MJ (2010). Propensity score estimation: neural networks, support vector machines, decision trees (CART), and meta-classifiers as alternatives to logistic regression. J Clin Epidemiol.

[8] Christodoulou E, Ma J, Collins GS, Steyerberg EW, Verbakel JY, Van Calster B (2019). A systematic review shows no performance benefit of machine learning over logistic regression for clinical prediction models. J Clin Epidemiol.

[9] Faisal M, Scally A, Howes R, Beatson K, Richardson D, Mohammed MA (2020). A comparison of logistic regression models with alternative machine learning methods to predict the risk of in-hospital mortality in emergency medical admissions via external validation. Health Informat J.

[10] Beunza J-J, Puertas E, García-Ovejero E, Villalba G, Condes E, Koleva G, Hurtado C, Landecho MF (2019). Comparison of machine learning algorithms for clinical event prediction (risk of coronary heart disease). J Biomed Inform.

[11] Sufriyana H, Husnayain A, Chen Y-L, Kuo C-Y, Singh O, Yeh T-Y, Wu Y-W, Su EC-Y (2020). Comparison of multivariable logistic regression and other machine learning algorithms for prognostic prediction studies in pregnancy care: systematic review and Meta-analysis. JMIR Med Inform.

[12] Jamthikar A, Gupta D, Saba L, Khanna NN, Araki T, Viskovic K, Mavrogeni S, Laird JR, Pareek G, Miner M (2020). Cardiovascular/stroke risk predictive calculators: a comparison between statistical and machine learning models. Cardiovasc Diagn Ther.

[13] Avuçlu E, Elen A (2020). Evaluation of train and test performance of machine learning algorithms and Parkinson diagnosis with statistical measurements. Med Biol Engi Comput.

[14] Belkin M, Hsu D, Ma S, Mandal S (2019). Reconciling modern machine-learning practice and the classical bias–variance trade-off. Proc Natl Acad Sci.

[15] Roelofs R, Shankar V, Recht B, Fridovich-Keil S, Hardt M, Miller J, Schmidt L (2019). A meta-analysis of overfitting in machine learning. Adv Neural Inf Proces Syst.

[16] Vollmer S, Mateen BA, Bohner G, Király FJ, Ghani R, Jonsson P, Cumbers S, Jonas A, McAllister KS, Myles P (2020). Machine learning and artificial intelligence research for patient benefit: 20 critical questions on transparency, replicability, ethics, and effectiveness. BMJ.

[17] Austin PC, Harrell FE, Steyerberg EW (2021). Predictive performance of machine and statistical learning methods: impact of data-generating processes on external validity in the “large N, small p” setting. Stat Methods Med Res.

[18] Desai RJ, Wang SV, Vaduganathan M, Evers T, Schneeweiss S (2020). Comparison of machine learning methods with traditional models for use of administrative claims with electronic medical records to predict heart failure outcomes. JAMA Netw Open.

[19] Bian J, Buchan I, Guo Y, Prosperi M (2019). Statistical thinking, machine learning. J Clin Epidemiol.

[20] Van Calster B (2019). Statistics versus machine learning: definitions are interesting (but understanding, methodology, and reporting are more important). J Clin Epidemiol.

[21] De Crescenzo F, Garriga C, Tomlinson A, Coupland C, Efthimiou O, Fazel S, Hippisley-Cox J, Cipriani A (2020). Real-world effect of antidepressants for depressive disorder in primary care: protocol of a population-based cohort study. Evid Based Mental Health.

[22] Kroenke K, Spitzer RL, Williams JB (2001). The PHQ-9: validity of a brief depression severity measure. J Gen Intern Med.

[23] Wahl I, Löwe B, Bjorner JB, Fischer F, Langs G, Voderholzer U, Aita SA, Bergemann N, Brähler E, Rose M (2014). Standardization of depression measurement: a common metric was developed for 11 self-report depression measures. J Clin Epidemiol.

[24] Leucht S, Fennema H, Engel RR, Kaspers-Janssen M, Szegedi A (2018). Translating the HAM-D into the MADRS and vice versa with equipercentile linking. J Affect Disord.

[25] Gillett G, Tomlinson A, Efthimiou O, Cipriani A (2020). Predicting treatment effects in unipolar depression: a meta-review. Pharmacol Ther.

[26] Džeroski S, Ženko B (2004). Is combining classifiers with stacking better than selecting the best one?. Mach Learn.

[27] Boehmke B, Greenwell BM (2019). Hands-on machine learning with R.

[28] Džeroski S, Ženko B (2002). Stacking with multi-response model trees. International Workshop on Multiple Classifier Systems.

[29] Polikar R (2006). Ensemble based systems in decision making. IEEE Circuits Syst Magaz.

[30] Wang H, Zheng B, Yoon SW, Ko HS (2018). A support vector machine-based ensemble algorithm for breast cancer diagnosis. Eur J Oper Res.

[31] Gashler M, Giraud-Carrier C, Martinez T (2008). Decision tree ensemble: small heterogeneous is better than large homogeneous. 2008 Seventh International Conference on Machine Learning and Applications.

[32] Altmann A, Toloşi L, Sander O, Lengauer T (2010). Permutation importance: a corrected feature importance measure. Bioinformatics.

[33] Breiman L (2001). Random forests. Mach Learn.

[34] Sterne JA, White IR, Carlin JB, Spratt M, Royston P, Kenward MG, Wood AM, Carpenter JR (2009). Multiple imputation for missing data in epidemiological and clinical research: potential and pitfalls. BMJ.

[35] Su Y-S, Gelman A, Hill J, Yajima M (2011). Multiple imputation with diagnostics (mi) in R: opening windows into the black box. J Stat Softw.

[36] Little RJ (1992). Regression with missing X’s: a review. J Am Stat Assoc.

[37] Lee KJ, Carlin JB (2012). Recovery of information from multiple imputation: a simulation study. Emerg Themes Epidemiol.

[38] Sullivan TR, Salter AB, Ryan P, Lee KJ (2015). Bias and precision of the “multiple imputation, then deletion” method for dealing with missing outcome data. Am J Epidemiol.

[39] Moons KG, Donders RA, Stijnen T, Harrell FE (2006). Using the outcome for imputation of missing predictor values was preferred. J Clin Epidemiol.

[40] Mustillo S, Kwon S (2015). Auxiliary variables in multiple imputation when data are missing not at random. J Mathemat Sociol.

[41] Kontopantelis E, White IR, Sperrin M, Buchan I (2017). Outcome-sensitive multiple imputation: a simulation study. BMC Med Res Methodol.

[42] Steyerberg EW, Harrell FE (2016). Prediction models need appropriate internal, internal–external, and external validation. J Clin Epidemiol.

[43] Takada T, Nijman S, Denaxas S, Snell KI, Uijl A, Nguyen T-L, Asselbergs FW, Debray TP (2021). Internal-external cross-validation helped to evaluate the generalizability of prediction models in large clustered datasets. J Clin Epidemiol.

[44] Acock AC (2008). A gentle introduction to Stata.

[45] Hmisc: Harrell Miscellaneous, package in R [https://cran.r-project.org/web/packages/Hmisc/index.html].

[46] Abadi M, Agarwal A, Barham P, Brevdo E, Chen Z, Citro C, Corrado GS, Davis A, Dean J, Devin M: TensorFlow: Large-scale machine learning on heterogeneous distributed systems. arXiv preprint arXiv:160304467 2016. 10.48550/arXiv.1603.04467.

[47] Raschka S (2015). Python machine learning.

[48] Bzdok D, Meyer-Lindenberg A (2018). Machine learning for precision psychiatry: opportunities and challenges. Biol Psychiatr.

[49] Rajula HSR, Verlato G, Manchia M, Antonucci N, Fanos V (2020). Comparison of conventional statistical methods with machine learning in medicine: diagnosis, drug development, and treatment. Medicina.

[50] Dong X, Yu Z, Cao W, Shi Y, Ma Q (2020). A survey on ensemble learning. Front Comput Sci.

[51] Harrell F (2019). Road map for choosing between statistical modeling and machine learning.

