# Supplementary material for: Development and validation of a meta-learner for combining statistical and machine learning prediction models in individuals with depression
Source: BMC Psychiatry. 2022 May 16;22:337. doi: 10.1186/s12888-022-03986-0 (PMC9112573; doi:10.1186/s12888-022-03986-0)
Supplement: Supplementary file 1 — Additional file 1. [file 12888_2022_3986_MOESM1_ESM.docx]

**Development and validation of a meta-learner for combining statistical and machine learning prediction models in individuals with depression – supplementary materials**

Contents

[Cohort Selection 2](#_Toc97993716)

[Predictors 6](#_Toc97993717)

[Descriptive Statistics of Study Cohort for the All-Cause Dropout Analysis (Table S1) 7](#_Toc97993718)

[Descriptive Statistics of Study Cohort for the Severity of Depressive Symptoms Analysis (Table S2) 10](#_Toc97993719)

[Mock Dataset (Table S3) 14](#_Toc97993720)

[Predictor Succinct Name Matching Table (Table S4) 16](#_Toc97993721)

[Multi-Layer Perceptron Implementation Details (Table S5) 18](#_Toc97993722)

[Statistical Differences of Outcomes between Missing and Non-Missing Values for Each Candidate Predictor (Table S6, Table S7) 19](#_Toc97993723)

[Bootstrap Task to Evaluate Performance of the Meta-Learners 21](#_Toc97993724)

[Internal-External Cross-Validation Implementation Details 21](#_Toc97993725)

[Predicting for New Patients in Practice 22](#_Toc97993726)

[Model Performance Analysis (Figure S1, Figure S2, Figure S3, Figure S4) 23](#_Toc97993727)

[Internal-External Evaluation (Table S8, Table S9) 27](#_Toc97993728)

[Feature Importance Analysis of the MLP Meta-Learner for PHQ-9 Prediction (Figure S5) 30](#_Toc97993729)

[Feature Importance Analysis of the MLP Meta-Learner for Dropout Prediction (Figure S6) 31](#_Toc97993730)

[Feature Importance Analysis of the MLP Base-Learner for PHQ-9 Prediction (Figure S7, Table S10) 32](#_Toc97993731)

[Feature Importance Analysis of the MLP Base-Learner for Dropout Prediction (Figure S8, Table S11) 35](#_Toc97993732)

[Feature Importance Analysis of the Linear Regression Meta-Learner for PHQ-9 Prediction (Figure S9) 38](#_Toc97993733)

[Feature Importance Analysis of the Logistic Regression Meta-Learner for Dropout Prediction (Figure S10) 39](#_Toc97993734)

[Feature Importance Analysis of the Ridge Regression Base-Learner for PHQ-9 Prediction (Figure S11, Table S12) 40](#_Toc97993735)

[Feature Importance Analysis of the Ridge Regression Base-Learner for Dropout Prediction (Figure S12, Table S13) 43](#_Toc97993736)

[Evaluation of Data Pre-Processing Techniques (Table S14) 46](#_Toc97993737)

[Reusing Base-Learner Predictors as Additional Predictors for the Meta-Learners (Table S15) 47](#_Toc97993738)

[References 48](#_Toc97993739)

# Cohort Selection

**Inclusion criteria**

We included patients registered with QResearch® with a recorded diagnosis of depression since 1^st^ Jan 1998. As in other studies [1-3], we used Read codes to identify cases of depression. A new Read code diagnosis of depression was considered a new episode of depression when preceded by 12 months of no depression diagnoses and no prescription of antidepressants. The index date for entry to the cohort was the date of depression diagnosis and patients were followed up for 3 months after the index date.

We considered 12 months of no diagnosis and no prescription of antidepressants as necessary to consider an episode of depression different from the eventual previous one (see below, in the exclusion criteria). This is because with treatment, episodes last on average 3 to 6 months, while most patients recover within 12 months [4] and in general practices the long-term course of depression is more favourable than in clinical samples [5].

Episodes of depression were included only if fluoxetine was prescribed within 12 days around the diagnosis of depression (i.e. 6 days before or after the diagnosis), because we were interested in the prognosis of patients treated with fluoxetine. Episodes where antidepressants were started 6 or more days prior to the index date of depression diagnosis were considered as if antidepressants were prescribed for other reasons than depression, and were excluded. Episodes with antidepressants prescribed after 6 days were considered as if patients were being on a watchful waiting/active monitoring and were excluded [6-8]

We did not use a specific threshold on a depression scale (e.g. PHQ-9 >5 at baseline) to include participants, as patients in primary care can be treated even with minor symptoms of depression [9] and GP ratings of severity do not always agree with validated screening instruments [10].

**Exclusion criteria**

We excluded:

- Episodes of depression which had a previous episode of depression in the year before or a previous prescription of antidepressants in the year before. This is because patients with multiple diagnoses and treatments within the year can be treatment-resistant and would therefore be a different population [11].
- Episodes of depression not associated with a prescription of an antidepressant at baseline (i.e., 6 days before or after the diagnosis).
- Episodes of depression associated with a prescription of more than one antidepressant in the year before (i.e. 365 days before or 6 days after the diagnosis);
- Episodes of depression associated with a prescription of antipsychotics in the year before (i.e. 365 days before or 6 days after the diagnosis);
- Episodes of depression associated with a prescription of mood stabilisers in the year before (i.e. 365 days before or 6 days after the diagnosis);
- Episodes of depression starting within 3 months of delivery (i.e. post-partum depression).

If a patient had multiple episodes of depression recorded, we gave preference to the last episode for each patient.

We also excluded patients with a recorded diagnosis of bipolar disorder or schizophrenia spectrum disorder made at any point before the index episode.

**Exposure**

The primary exposure of interest was the use of fluoxetine. Information was extracted from all prescriptions for fluoxetine issued during the 3-months follow-up. We calculated the duration of each prescription in days by dividing the number of tablets prescribed by the number of tablets to be taken each day. If the information on tablets per day was missing or not sufficiently detailed (expected to be < 5% of total prescriptions) we estimated the duration of the prescription based on the number of tablets prescribed, as in previous studies [3]. Patients were classified as continually exposed to fluoxetine during periods where there were no gaps of more than 30 days between the end of one prescription and the start of the next (most antidepressants at the beginning of a treatment are prescribed for not more than 28-30 days). Patients were classified as exposed for the first 30 days after the estimated date of stopping fluoxetine in order to account for any delays in starting the prescription or accumulation of tablets as well as to attribute the outcomes occurring during withdrawal periods to the antidepressant, as done in previous studies.

# Predictors

Demographic variables included age at diagnosis (continuous), sex (male or female), Townsend quintiles (from 1: least deprived to 5: most deprived), ethnicity (White/Caucasian, African/Caribbean, Asian, Other), Body Mass Index (BMI, continuous), smoking status (currently non-smoker, smoker).

Condition-specific variables included baseline depression severity (continuous, we considered PHQ-9 recorded up to two weeks before and 6 days after the index diagnosis of depression as the baseline measurement), previous antidepressant use (yes/no), use of selective serotonin reuptake inhibitor (SSRI) in the past (yes/no), use of fluoxetine in the past (yes/no), previous psychotherapy use (yes/no), previous referral to secondary care (yes/no), childhood maltreatment (yes/no). Note that the use of medications would be more than a year before, exactly. Episodes where patients were prescribed antidepressants in the year before were excluded.

Comorbid conditions included coronary heart disease, stroke/transient ischemic attack, diabetes, epilepsy/seizures, hypothyroidism, chronic inflammatory diseases (including Osteoarthritis and Rheumatoid arthritis), anxiety or obsessive-compulsive disorder, migraine.

Use of other drugs at baseline included antihypertensive drugs, aspirin, statins, anticoagulants, non-steroidal anti-inflammatory drugs, anticonvulsants, hypnotics/anxiolytics, bisphosphonates, oral contraceptives or hormone replacement therapy.

# Descriptive Statistics of Study Cohort for the All-Cause Dropout Analysis (Table S1)

Table S1. Descriptive statistics of study cohort for the all-cause dropout analysis (N=187,757). N: number. SD: standard deviation.

| **Characteristic** | **Sample**  N (%) / mean (SD) | **Missing**  N (%) |
| --- | --- | --- |
| Predictors - Demographic |  |  |
| Sex |  | 0 |
| Female | 119,169 (63.47) |  |
| Male | 68,588 (36.53) |  |
| Age | 42.99 (15.93) | 0 |
| Body Mass Index (BMI) | 26.74 (6.23) | 36,345 (19.36) |
| Smoking |  | 12,915 (6.88) |
| Yes | 60,120 (32.02) |  |
| No | 114,722 (61.10) |  |
| Ethnic group |  | 56,039 (29.85) |
| White | 119,804 (63.81) |  |
| African/Caribbean | 2,784 (1.48) |  |
| Asian | 5,667 (3.02) |  |
| Other | 3,463 (1.84) |  |
| Townsend deprivation score |  | 545 (0.29) |
| 1 (Least deprived) | 42,252 (22.50) |  |
| 2 | 43,799 (23.33) |  |
| 3 | 40,693 (21.67) |  |
| 4 | 34,287 (18.26) |  |
| 5 (Most deprived) | 26,181 (13.94) |  |
| Predictors - Depression specific |  |  |
| Baseline Patient Health Questionnaire (PHQ-9) | 17.19 (4.90) | 141,575 (75.40) |
| First episode |  | 0 |
| Yes | 136,263 (72.57) |  |
| No | 51,494 (27.43) |  |
| Previous antidepressant use |  | 0 |
| Any antidepressant | 63,797 (33.98) |  |
| Any selective serotonin reuptake inhibitor (SSRI) | 51,041 (27.18) |  |
| Fluoxetine | 36,692 (19.54) |  |
| Previous psychotherapy | 1771 (0.94) | 0 |
| Previous referral to secondary care | 783 (0.42) | 0 |
| Childhood maltreatment | 279 (0.15) | 0 |
| Predictors - Comorbid conditions at baseline |  |  |
| Coronary heart disease | 6,024 (3.21) | 0 |
| Stroke | 3,853 (2.05) | 0 |
| Diabetes | 8,429 (4.49) | 0 |
| Epilepsy | 2,253 (1.20) | 0 |
| Hypothyroidism | 6,548 (3.49) | 0 |
| Arthritis | 13,046 (6.95) | 0 |
| Anxiety | 27,075 (14.42) | 0 |
| Migraine | 14,367 (7.65) | 0 |
| Predictors - Use of other drugs at baseline |  |  |
| Antihypertensive | 13,919 (7.41) | 0 |
| Aspirin | 7,472 (3.98) | 0 |
| Statins | 12,118 (6.45) | 0 |
| Anticoagulants | 1,578 (0.84) | 0 |
| Non-steroidal anti-inflammatory drugs (NSAIDs) | 7,912 (4.21) | 0 |
| Anticonvulsants | 2,479 (1.32) | 0 |
| Hypnotics | 13,244 (7.05) | 0 |
| Bisphosphonates | 518 (0.28) | 0 |
| Contraceptives | 12,536 (6.68) | 0 |
| Outcomes |  |  |
| PHQ-9 at 2 months | 11.70 (6.34) | 171,373 (91.27) |
| Discontinuation due to any cause |  | 0 |
| Dropouts | 70,406 (37.5) |  |
| Retention in treatment | 117,351 (62.5) |  |

# Descriptive Statistics of Study Cohort for the Severity of Depressive Symptoms Analysis (Table S2)

Table S2. Descriptive statistics of study cohort for the severity of depressive symptoms analysis. (N=16,384). N: number. SD: standard deviation.

| **Characteristic** | **Sample**  **N (percentage) / mean (SD)** | **Missing**  **N (percentage)** |
| --- | --- | --- |
| Predictors - Demographic |  |  |
| Sex |  | 0 |
| Female | 10,226 (62.41) |  |
| Male | 6,158 (37.59) |  |
| Age | 41.99 (14.20) | 0 |
| Body Mass Index (BMI) | 27.34 (6.41) | 2,184 (13.33) |
| Smoking |  | 236 (1.44) |
| Yes | 4,944 (30.18) |  |
| No | 11,204 (68.38) |  |
| Ethnic group |  | 2,438 (14.88) |
| White | 12,834 (78.33) |  |
| African/Caribbean | 223 (1.36) |  |
| Asian | 544 (3.32) |  |
| Other | 345 (2.11) |  |
| Townsend deprivation score |  | 38 (0.23) |
| 1 (Least deprived) | 3,816 (23.29) |  |
| 2 | 3,923 (23.94) |  |
| 3 | 3,538 (21.59) |  |
| 4 | 2,935 (17.91) |  |
| 5 (Most deprived) | 2,134 (13.02) |  |
| Predictors - Depression specific |  |  |
| Baseline Patient Health Questionnaire (PHQ-9) | 17.45 (4.74) | 3,630 (22.16) |
| First episode |  | 0 |
| Yes | 11,018 (67.25) |  |
| No | 5,366 (32.75) |  |
| Previous antidepressant use |  | 0 |
| Any antidepressant | 6,290 (38.39) |  |
| Any selective serotonin reuptake inhibitor (SSRI) | 5,196 (31.71) |  |
| Fluoxetine | 3,781 (23.08) |  |
| Previous psychotherapy | 151 (0.92) | 0 |
| Previous referral to secondary care | 56 (0.34) | 0 |
| Childhood maltreatment | 24 (0.15) | 0 |
| Predictors - Comorbid conditions at baseline |  |  |
| Coronary heart disease | 423 (2.58) | 0 |
| Stroke | 253 (1.54) | 0 |
| Diabetes | 827 (5.05) | 0 |
| Epilepsy | 186 (1.14) | 0 |
| Hypothyroidism | 627 (3.83) | 0 |
| Arthritis | 1,188 (7.25) | 0 |
| Anxiety | 2,623 (16.01) | 0 |
| Migraine | 1,529 (9.33) | 0 |
| Predictors - Use of other drugs at baseline |  |  |
| Antihypertensive | 1,426 (8.70) | 0 |
| Aspirin | 593 (3.62) | 0 |
| Statins | 1,327 (8.10) | 0 |
| Anticoagulants | 106 (0.65) | 0 |
| Non-steroidal anti-inflammatory drugs (NSAIDs) | 632 (3.86) | 0 |
| Anticonvulsants | 219 (1.34) | 0 |
| Hypnotics | 1,214 (7.41) | 0 |
| Bisphosphonates | 44 (0.27) | 0 |
| Contraceptives | 1,026 (6.26) | 0 |
| Outcomes |  |  |
| PHQ-9 at 2 months | 11.70 (6.34) | 0 |

# Mock Dataset (Table S3)

Table S3. Mock dataset of five patient.

| **Predictor/Outcome** | **Patient 1** | **Patient 2** | **Patient 3** | **Patient 4** | **Patient 5** |
| --- | --- | --- | --- | --- | --- |
| Sex | Female | Male | Female | Male | Female |
| Age | 43 | 59 | 71 | 26 | 34 |
| Body Mass Index (BMI) | 30.23 | 32.89 | 27.71 | 21.90 | 19.31 |
| Smoking | Yes | No | No | No | No |
| Ethnic group | White | Asian | White | White | Other |
| Townsend deprivation score | 1 | 2 | 2 | 4 | 5 |
| Baseline Patient Health Questionnaire (PHQ-9) | 21 | 23 | 17 | 18 | 19 |
| First episode | Yes | Yes | Yes | No | No |
| Use of antidepressant | No | No | No | Yes | No |
| Use of any selective serotonin reuptake inhibitor (SSRI) | No | No | No | Yes | No |
| Use of fluoxetine | No | No | No | No | No |
| Previous psychotherapy | No | No | No | No | Yes |
| Previous referral to secondary care | No | No | No | Yes | No |
| Childhood maltreatment | No | No | Yes | No | No |
| Coronary heart disease | No | No | No | No | Yes |
| Stroke | No | No | Yes | No | No |
| Diabetes | No | No | Yes | No | No |
| Epilepsy | No | Yes | No | No | No |
| Hypothyroidism | No | No | No | No | Yes |
| Arthritis | No | No | No | No | Yes |
| Anxiety | No | No | Yes | No | No |
| Migraine | Yes | No | No | No | No |
| Antihypertensive | No | No | Yes | No | No |
| Aspirin | Yes | No | No | No | No |
| Statins | No | No | No | No | No |
| Anticoagulants | Yes | No | No | No | No |
| Non-steroidal anti-inflammatory drugs (NSAIDs) | No | No | No | No | No |
| Anticonvulsants | No | Yes | No | No | No |
| Hypnotics | No | No | No | Yes | No |
| Bisphosphonates | No | No | No | No | No |
| Contraceptives | No | No | No | No | Yes |
| Dropout | No | No | Yes | Yes | Yes |
| PHQ-9 at 2 months | 11 | 21 | 10 | 7 | 3 |

# Predictor Succinct Name Matching Table (Table S4)

Table S4. Predictor succinct name matching table.

| **Predictor succinct name** | **Predictor description** |
| --- | --- |
| sex1 | Sex (male or female) |
| first_ep | First episode |
| flx_past | Fluoxetine used in the past |
| SSRI_past | Selective serotonin reuptake inhibitor (SSRI) used in the past |
| AD_past | Antidepressant used in the past |
| bmi | Body mass index |
| smoker | Currently smoker or non-smoker |
| PHQ9_base | Patient Health Questionnaire (PHQ-9) baseline depression severity |
| past_psychotherapy | Previous psychotherapy use |
| past_referral | Previous referral to secondary care |
| past_maltreatment | Childhood maltreatment |
| chd | Comorbid condition - coronary heart disease |
| stroke | Comorbid condition - stroke/transient ischaemic attack |
| diabetes | Comorbid condition - diabetes |
| epilepsy | Comorbid condition - epilepsy/seizures |
| hypothyroidism | Comorbid condition - hypothyroidism |
| arthritis | Comorbid condition - chronic inflammatory diseases (including Osteoarthritis and Rheumatoid arthritis) |
| anxiety | Comorbid condition - anxiety or Obsessive-compulsive disorder |
| migraine | Comorbid condition - migraine |
| antihypertensive | Use of antihypertensive drugs at baseline |
| aspirin | Use of aspirin at baseline |
| statins | Use of statins at baseline |
| anticoagulants | Use of anticoagulants at baseline |
| NSAIDs | Use of non-steroidal anti-inflammatory drugs at baseline |
| anticonvulsants | Use of anticonvulsants at baseline |
| hypnotics | Use of hypnotics/anxiolytics at baseline |
| bisphosphonates | Use of bisphosphonates at baseline |
| contraceptives | Use of oral contraceptives or hormone replacement therapy at baseline |
| age_current | Age at diagnosis |
| ethrisk_PA | Ethnicity (white/Caucasian, African/Caribbean, Asian, Other) |
| town_quintile | Townsend quintiles (from 1-least deprived to 5-most deprived) |
| pred_ml | machine learning base-learner predictions |
| pred_st | statistical base-learner predictions |

# Multi-Layer Perceptron Implementation Details (Table S5)

Table S5. Implementation details of the multi-layer perceptron (MLP) model.

| Structure | Fully connected layer (FC1) + FC2 + FC3 + FC4 |
| --- | --- |
| Hyperparameter | FC1, FC2, FC3 units: 256  FC1, FC2, FC3 activation function: tanh  FC1, FC2, FC3 dropout: 0.2  FC4 units: 1  FC4 activation function: sigmoid for dropout, relu for PHQ-9  Learning rate: 0.001  Optimiser: Adam  Loss function: binary cross entropy for dropout, mean absolute error for PHQ-9  Early stopping monitor: area under the receiver operating characteristic curve for dropout, mean absolute error for PHQ-9  Early stopping patience: 50 |

# Statistical Differences of Outcomes between Missing and Non-Missing Values for Each Candidate Predictor (Table S6, Table S7)

Table S6. Testing for the difference of Patient Health Questionnaire (PHQ-9) score (mean [standard deviation]) at 2 months between missing and non-missing values for each candidate predictor. t: T-test. P: p-value.

|  | Non-missing | Missing | Test results |
| --- | --- | --- | --- |
| Body Mass Index | 11.74 [6.41]  N = 151,412 | 12.02 [6.47]  N = 36,345 | $t\left( 54732.36 \right)=-7.39$  $P<0.01$ |
| Smoking | 11.79 [6.41]  N = 174,842 | 11.92 [6.52]  N = 12,915 | $t\left( 14819.83 \right)=-2.28$  $P=0.02$ |
| Ethnicity group | 11.84 [6.42]  N = 131,718 | 11.68 [6.41]  N = 56,039 | $t\left( 105855.46 \right)=4.97$  $P<0.01$ |
| Townsend deprivation score | 11.89 [6.42]  N = 187,212 | 11.97 [6.62]  N = 545 | $t\left( 546.98 \right)=-0.62$  $P=0.53$ |
| Baseline PHQ-9 | 11.63 [6.36]  N = 46,182 | 11.85 [6.44]  N = 141,575 | $t\left( 79275.65 \right)=-6.57$  $P<0.01$ |

Table S7. Testing for the difference of dropout (%) between missing and non-missing values for each candidate predictor. $\chi^{2}$: Chi-square test. P: p-value.

|  | Non-missing | Missing | Test results |
| --- | --- | --- | --- |
| Body Mass Index | 36.85  N = 151,412 | 40.20  N = 36,345 | $\chi^{2}\left( 1 \right)=140.58, P<0.01$ |
| Smoking | 37.17  N = 174,842 | 41.90  N = 12,915 | $\chi^{2}\left( 1 \right)=114.29, P<0.01$ |
| Ethnicity group | 36.61  N = 131,718 | 39.59  N = 56,039 | $\chi^{2}\left( 1 \right)=149.01, P<0.01$ |
| Townsend deprivation score | 37.49  N = 187,212 | 41.10  N = 545 | $\chi^{2}\left( 1 \right)=2.87, P=0.08$ |
| Baseline PHQ-9 | 34.47  N = 46,182 | 38.49  N = 141,575 | $\chi^{2}\left( 1 \right)=239.14, P<0.01$ |

# Bootstrap Task to Evaluate Performance of the Meta-Learners

To estimate the variability in performance measures for the meta-learners, we performed an additional bootstrapping task, where we generated a bootstrap sample on the meta-learner predictors, i.e., the base-learner out-of-sample predictions. Next, we fitted the linear/logistic regression and MLP meta-learners separately on the bootstrap sample and made predictions on out-of-samples. We then calculated measures of performance for each meta-learner. We repeated this 200 times.

# Internal-External Cross-Validation Implementation Details

We used 10 regions of England, where the data were collected, as an auxiliary variable for geographical train test split. Specifically, we took samples from randomly selected 9 regions to develop base- and meta-learners and tested all models on the left-out region. In terms of the base-learners, we directly used all samples from these 9 regions for training and tested them on the left-out region. To prepare the training data for the meta-learners, we adopted the previous strategy [12, 13], where we deployed 10-fold cross validation and fitted the base-learners on samples from these 9 regions only. The out-of-fold predictions were then compiled as the training data for the meta-learners. Next, we developed the meta-learners on these predictions made by the base-learners and tested the meta-learners on the left-out region. To evaluate the uncertainty around the measures of performance, we applied the same strategy on the 10 imputed datasets. We then selected another region as the left-out region and tested all models. Finally, we reported the measures of performance per region and the average.

# Predicting for New Patients in Practice

To use a meta-learner to predict for a new patient we can follow the approach we described in the manuscript. We create a large number of bootstrap samples (e.g., 200), train all base-learners in each sample, and use the average of out-of-sample predictions for each patient and for each base-learner as inputs for developing the meta-learner. Then, for a new patient we use the 200 already developed sets of base-learners to create predictions, we average them and use them as input to the already developed meta-learner. Note that instead of a bootstrap method we could use the leave-one-patient-out approach.

For big datasets and when we use many complicated base-learners, this strategy may be computationally infeasible. In that case, k-fold cross validation can be used as an alternative. More specifically, we first split the data into k folds, use k-1 folds to train all base-learners and make predictions on the remaining fold. We then cycle through all folds. In the end, each patient obtains a single prediction from each base-learner. We then develop the meta-learner based on these predictions. Finally, to predict for a new patient we use the already developed k sets of base-learners to obtain k predictions for each base-learner; we average and use them as inputs for the already developed meta-learner to get the final prediction. Alternatively, we can retrain the base-learners on the whole dataset and obtain two new base-learners only and feed the predictions from these two base-learners to the already developed meta-learner to get the final prediction.

# Model Performance Analysis (Figure S1, Figure S2, Figure S3, Figure S4)


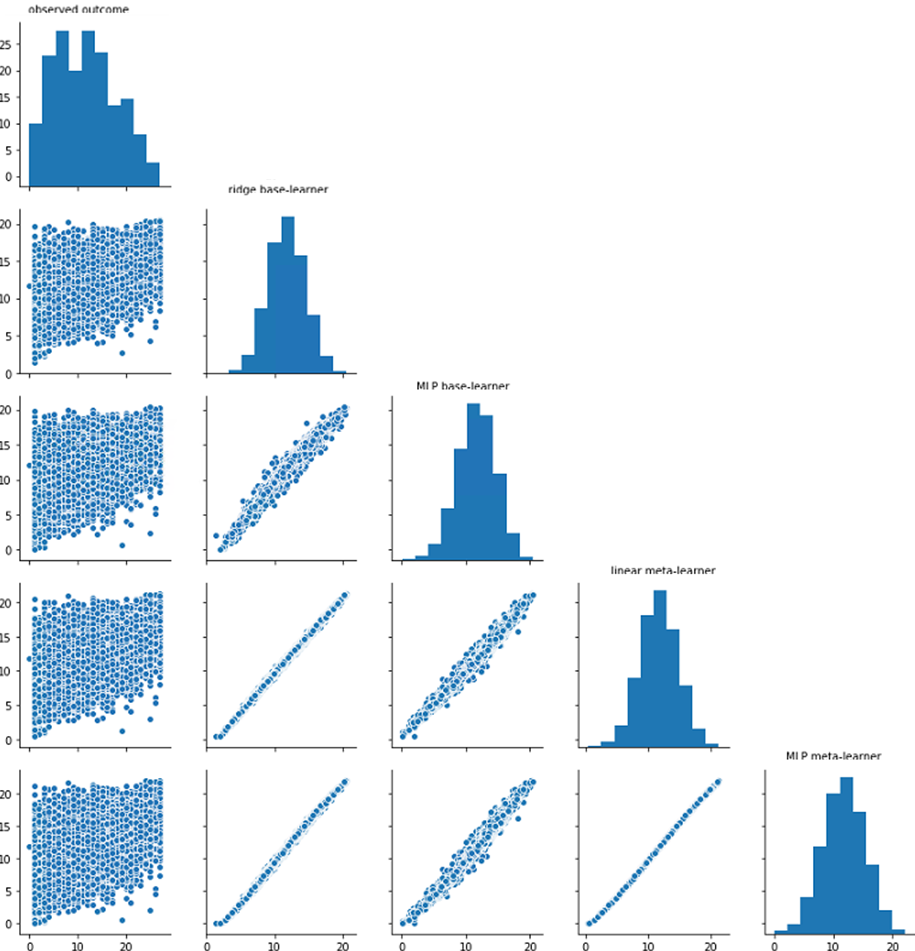


Figure S1. Pair plots of the predicted Patient Health Questionnaire (PHQ-9) scores of the base- and meta-learners. MLP: multi-layer perceptron.

A

B

C

D


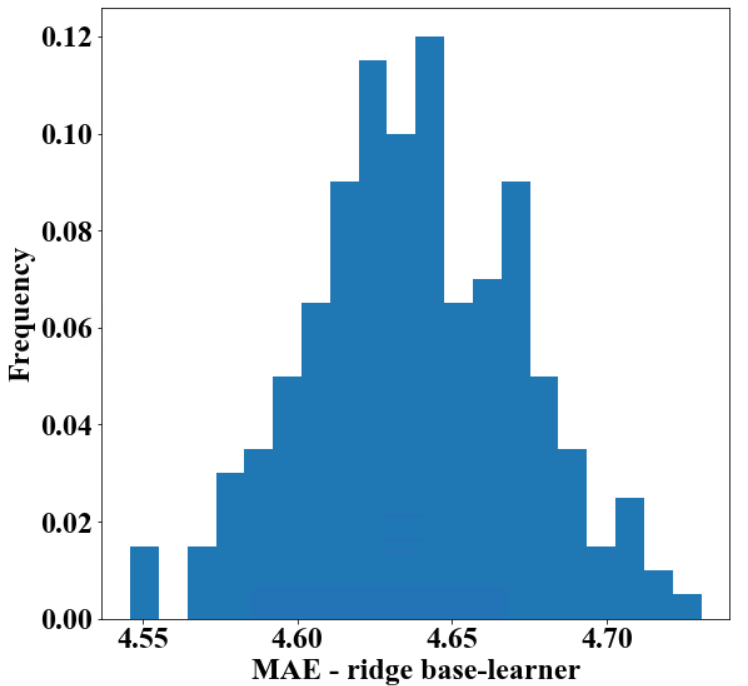

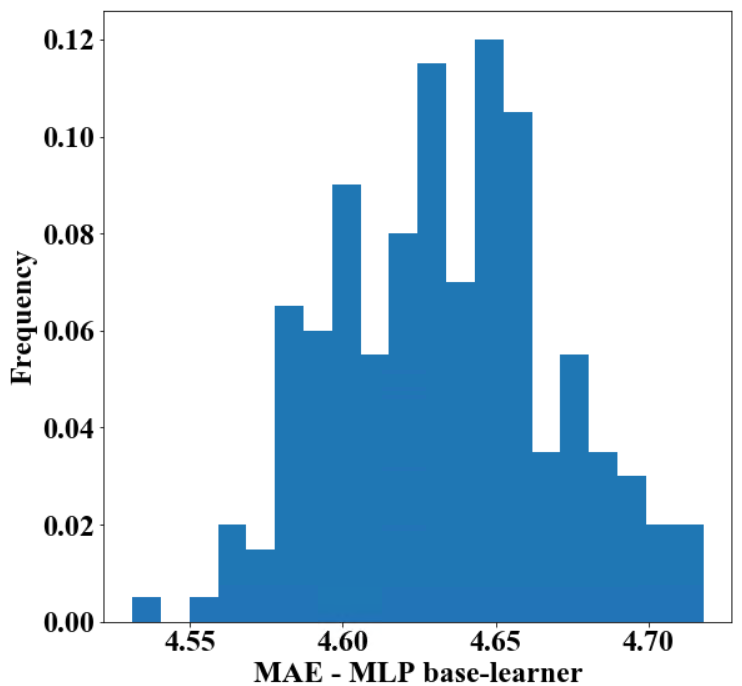

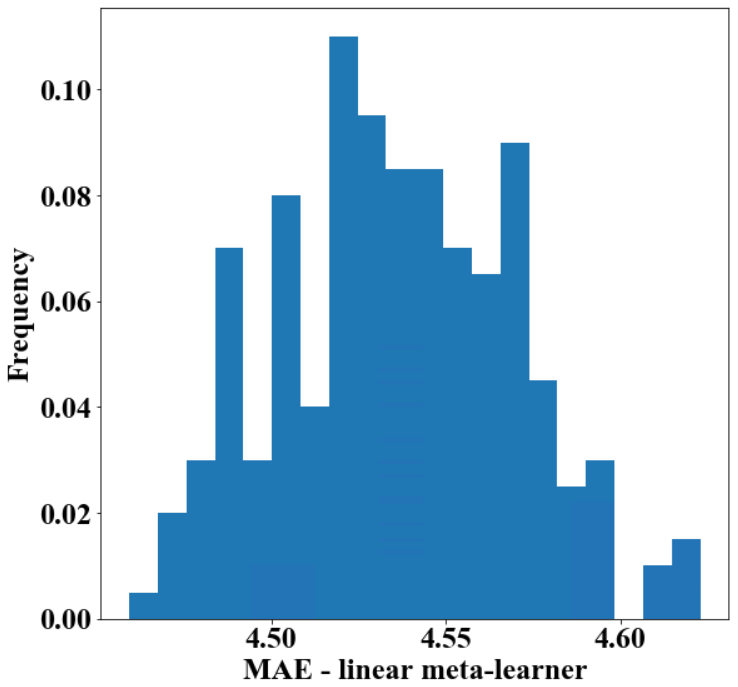

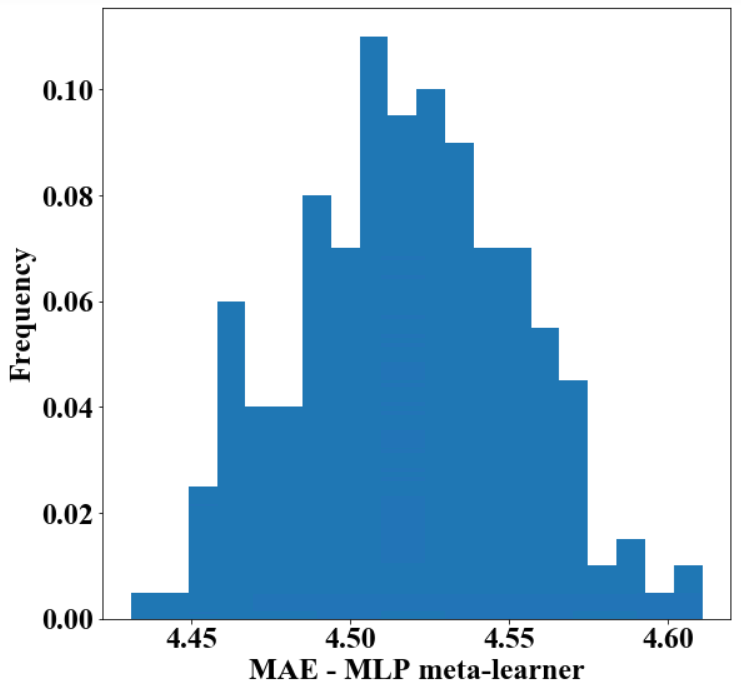


Figure S2. Histograms of the mean absolute errors (MAEs) for Patient Health Questionnaire-9 (PHQ-9) score prediction, 200 bootstrap samples. (A) Ridge regression base-learner. (B) Multi-layer perceptron (MLP) base-learner. (C) Linear regression meta-learner without regularization. (D) MLP meta-learner.


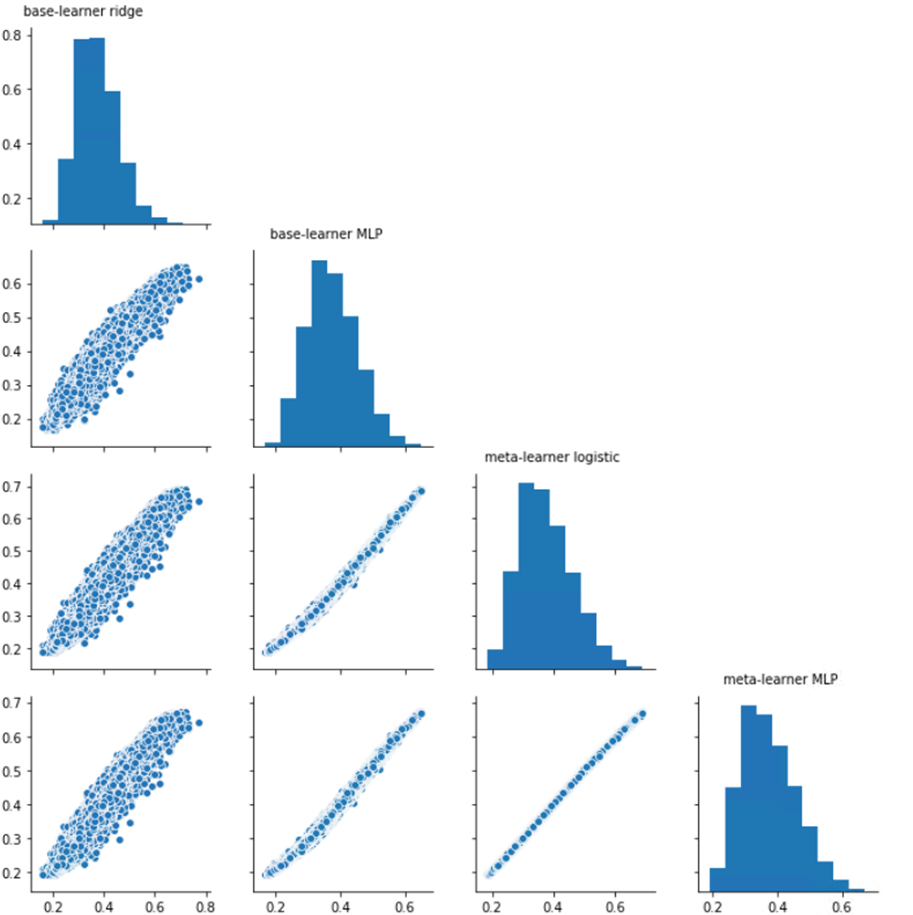


Figure S3. Pair plots of the predicted probabilities of dropout of the base- and meta-learners. MLP: multi-layer perceptron.

A

B

C

D


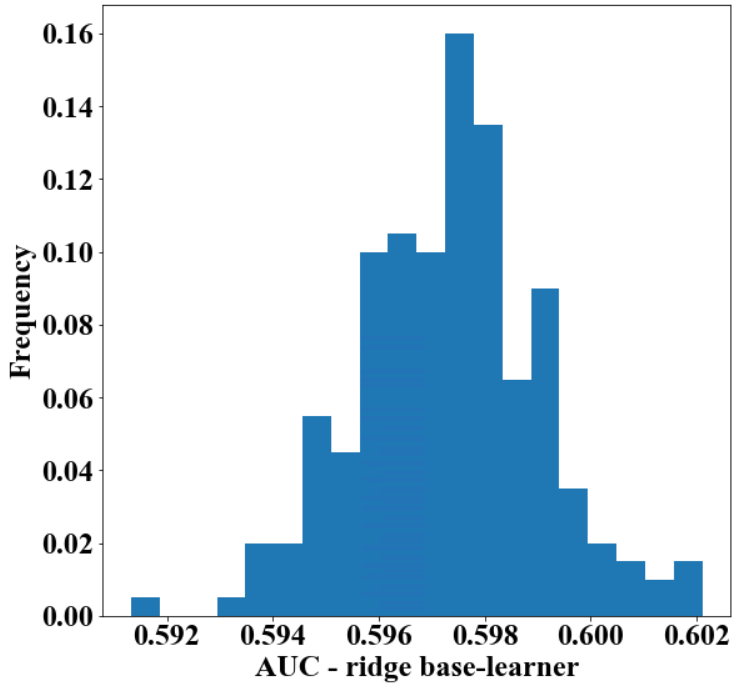

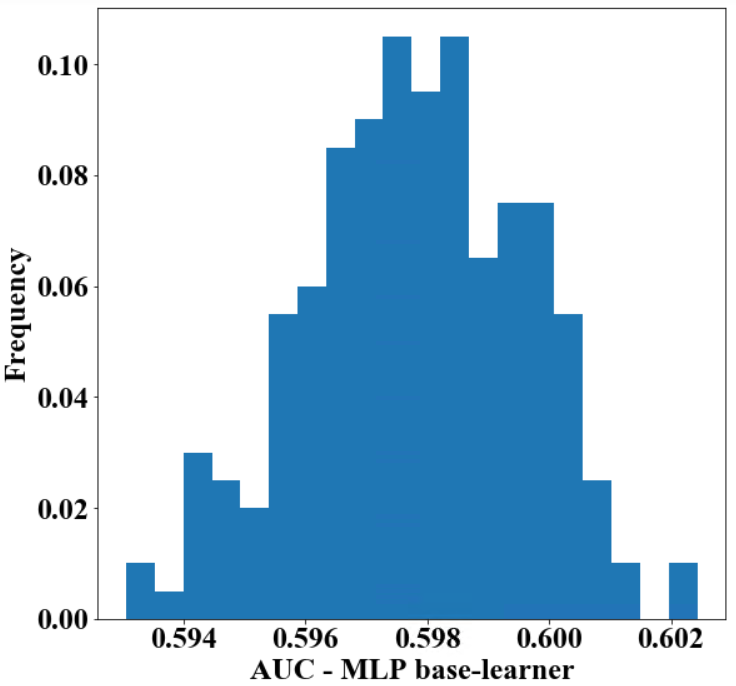

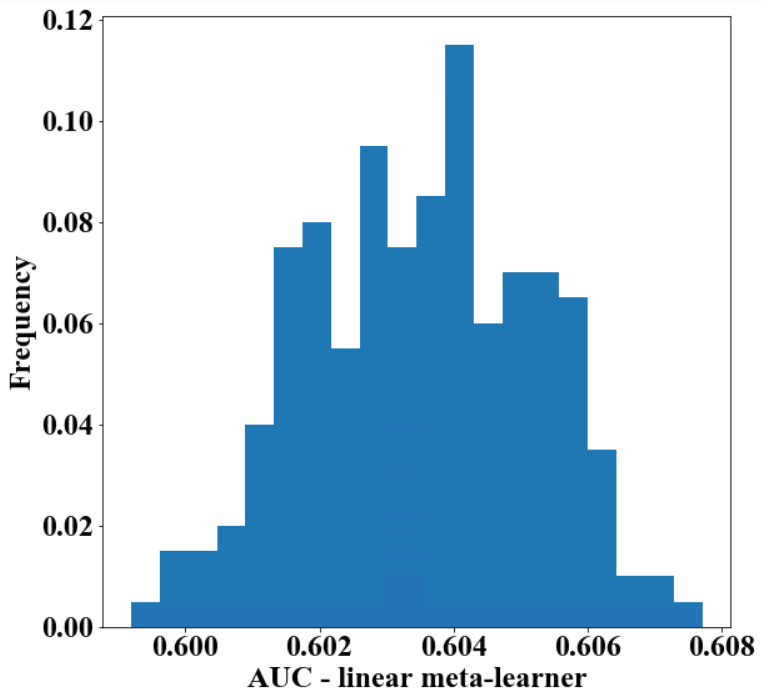

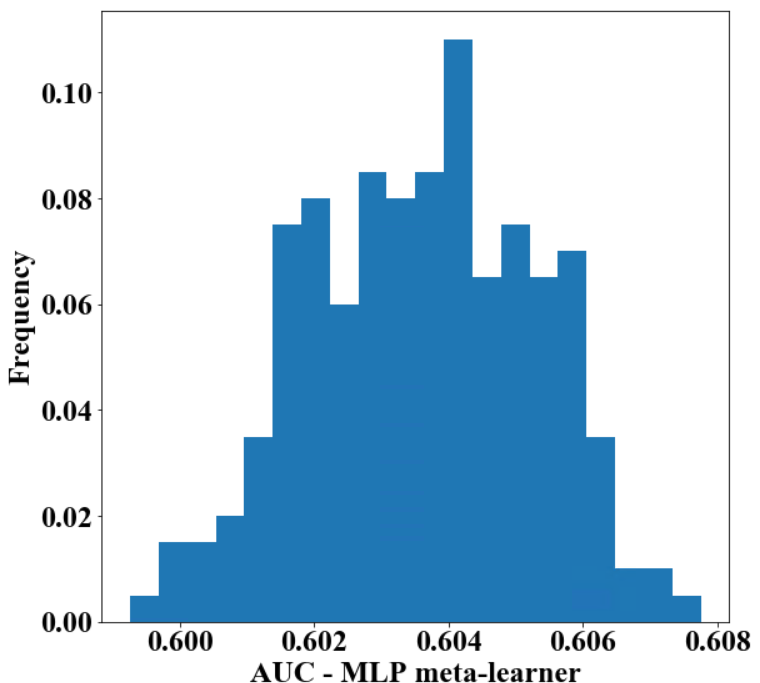


Figure S4. Histograms of the area under the receiver operating characteristic curve (AUC) for dropout prediction, 200 bootstrap samples. (A) Ridge regression base-learner. (B) Multi-layer perceptron (MLP) base-learner. (C) Logistic regression meta-learner without regularization. (D) MLP meta-learner.

# Internal-External Evaluation (Table S8, Table S9)

Table S8. Internal_external evaluation on post treatment Patient Health Questionnaire (PHQ-9) predictions (MAE [95% CI]) made by the models. MLP: multi-layer perceptron. MAE: mean absolute error. CI: confidence interval, calculated by interval from the 2.5^th^ to the 97.5^th^ percentile.

| Region | Base-learner ridge regression | Base-learner MLP | Meta-learner linear/logistic regression | Meta-learner MLP |
| --- | --- | --- | --- | --- |
| East Midlands  N=616 (3.76%) | 4.658 [4.611, 4.692] | 4.684 [4.638, 4.722] | 4.574 [4.529, 4.608] | 4.615 [4.602, 4.624] |
| East of England  N=662 (4.04%) | 4.518 [4.473, 4.580] | 4.533 [4.477, 4.600] | 4.423 [4.374, 4.486] | 4.433 [4.423, 4.440] |
| London  N=2,725 (16.63%) | 4.637 [4.611, 4.659] | 4.633 [4.604, 4.661] | 4.551 [4.526, 4.573] | 4.505 [4.499, 4.513] |
| North East  N=973 (5.94%) | 4.909 [4.879, 4.938] | 4.877 [4.842, 4.903] | 4.832 [4.803, 4.860] | 4.727 [4.713, 4.743] |
| North West  N=3,023 (18.45%) | 4.668 [4.647, 4.699] | 4.673 [4.652, 4.713] | 4.578 [4.558, 4.608] | 4.550 [4.544, 4.555] |
| South Central  N=2,497 (15.24%) | 4.588 [4.556, 4.614] | 4.583 [4.554, 4.610] | 4.500 [4.469, 4.527] | 4.510 [4.502, 4.516] |
| South East  N=1,440 (8.79%) | 4.605 [4.575, 4.635] | 4.626 [4.590, 4.659] | 4.516 [4.486, 4.546] | 4.558 [4.541, 4.570] |
| South West  N=1,706 (10.41%) | 4.573 [4.541, 4.615] | 4.556 [4.524, 4.607] | 4.486 [4.455, 4.530] | 4.446 [4.439, 4.454] |
| West Midlands  N=1,657 (10.11%) | 4.776 [4.740, 4.805] | 4.774 [4.773, 4.809] | 4.689 [4.653, 4.719] | 4.704 [4.692, 4.710] |
| Yorkshire & Humber  N=1,085 (6.62%) | 4.391 [4.347, 4.444] | 4.390 [4.333, 4.446] | 4.297 [4.252, 4.349] | 4.230 [4.220, 4.240] |
| Average  N=16,384 | 4.636 [4.619, 4.647] | 4.634 [4.619, 4.649] | 4.548 [4.531, 4.560] | 4.529 [4.526, 4.532] |

Table S9. Internal-external evaluation on all-cause treatment dropout predictions (AUC [95% CI]) made by the models. MLP: multi-layer perceptron. AUC: area under the receiver operating characteristic curve. CI: confidence interval, calculated by interval from the 2.5^th^ to the 97.5^th^ percentile.

| Region | Base-learner ridge regression | Base-learner MLP | Meta-learner linear/logistic regression | Meta-learner MLP |
| --- | --- | --- | --- | --- |
| East Midlands  N=7,693 (4.10%) | 0.587 [0.583, 0.592] | 0.589 [0.585, 0.592] | 0.594 [0.594, 0.594] | 0.595 [0.594, 0.597] |
| East of England  N=7,766 (4.14%) | 0.588 [0.596, 0.592] | 0.597 [0.594, 0.599] | 0.602 [0.602, 0.602] | 0.602 [0.601, 0.603] |
| London  N=31,530 (16.79%) | 0.600 [0.599, 0.601] | 0.596 [0.594, 0.597] | 0.603 [0.603, 0.603] | 0.601 [0.600, 0.602] |
| North East  N=9,669 (5.15%) | 0.580 [0.578, 0.582] | 0.586 [0.583, 0.588] | 0.590 [0.590, 0.590] | 0.590 [0.589, 0.591] |
| North West  N=35,757 (19.04%) | 0.597 [0.595, 0.599] | 0.601 [0.599, 0.602] | 0.606 [0.606, 0.606] | 0.607 [0.606, 0.607] |
| South Central  N=28,404 (15.13%) | 0.595 [0.593, 0.596] | 0.597 [0.595, 0.598] | 0.602 [0.602, 0.602] | 0.603 [0.602, 0.603] |
| South East  N=16,369 (8.72%) | 0.586 [0.584, 0.588] | 0.589 [0.587, 0.590] | 0.593 [0.593, 0.593] | 0.594 [0.593, 0.595] |
| South West  N=21,146 (11.26%) | 0.588 [0.587, 0.589] | 0.595 [0.594, 0.596] | 0.600 [0.600, 0.600] | 0.601 [0.600, 0.602] |
| West Midlands  N=17,659 (9.41%) | 0.600 [0.599, 0.602] | 0.601 [0.600, 0.602] | 0.606 [0.606, 0.606] | 0.605 [0.605, 0.606] |
| Yorkshire & Humber  N=11,764 (6.27%) | 0.586 [0.584, 0.589] | 0.584 [0.582, 0.587] | 0.588 [0.588, 0.588] | 0.587 [0.587, 0.588] |
| Average  N=187,757 | 0.597 [0.596, 0.597] | 0.598 [0.597, 0.599] | 0.603 [0.603, 0.603] | 0.603 [0.603, 0.603] |

# Feature Importance Analysis of the MLP Meta-Learner for PHQ-9 Prediction (Figure S5)


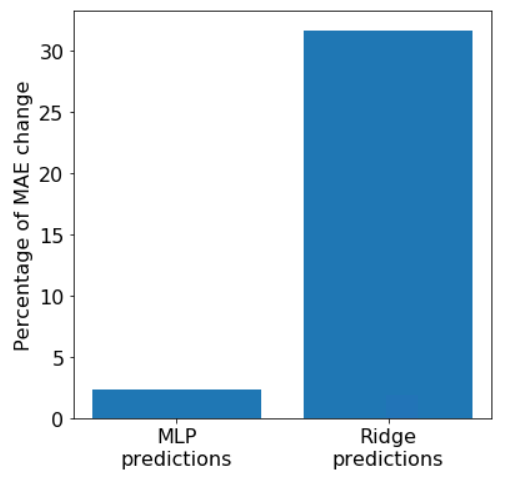


Figure S5. Permutation feature importance of the multi-layer perceptron (MLP) meta-learner for Patient Health Questionnaire (PHQ-9) prediction, evaluated on the meta-learner using base-learner predictions only as input. The x-axis represents pre-defined predictors and the y-axis represents the percentage increase of mean absolute error (MAE). Each feature was shuffled 100 times.

# Feature Importance Analysis of the MLP Meta-Learner for Dropout Prediction (Figure S6)


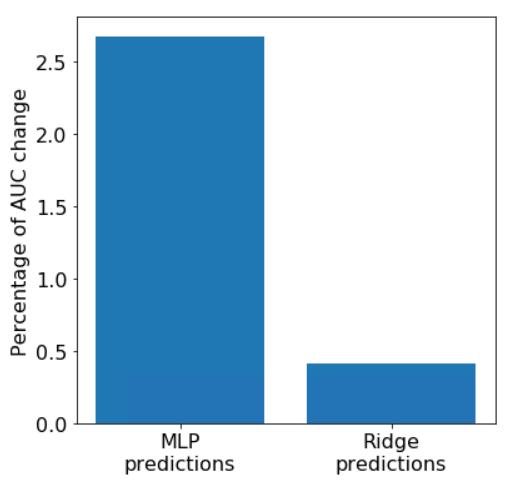


Figure S6. Permutation feature importance of the multi-perceptron (MLP) meta-learner for all-cause treatment dropout prediction, evaluated on the meta-learner using base-learner predicted probabilities only as input. The x-axis represents pre-defined predictors and the y-axis represents the percentage decrease of area under the receiver operating characteristic curve (AUC). Each feature was shuffled 100 times.

# Feature Importance Analysis of the MLP Base-Learner for PHQ-9 Prediction (Figure S7, Table S10)


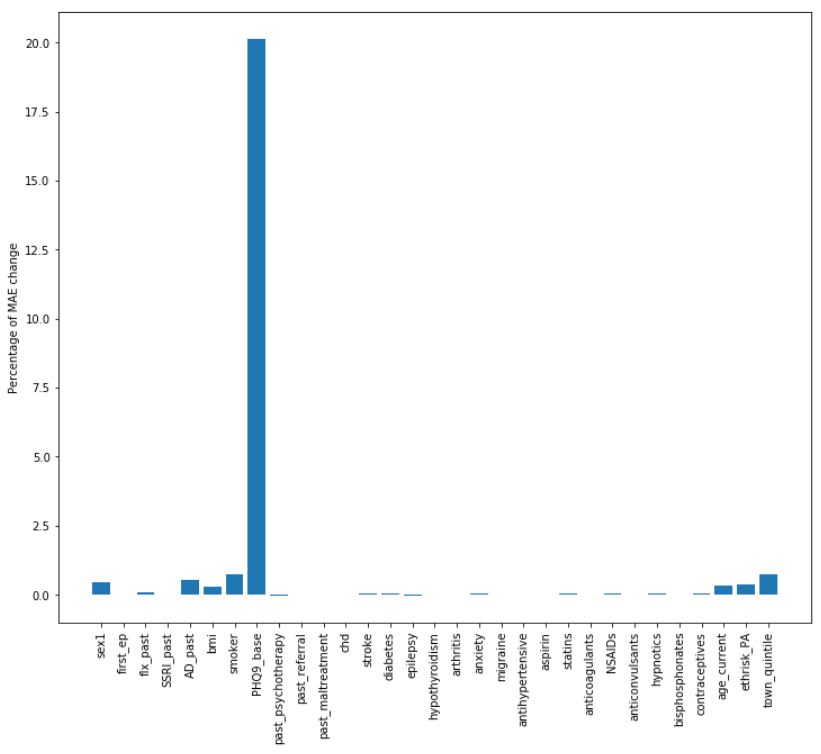


Figure S7. Permutation feature importance of the multi-layer perceptron (MLP) base-learner for Patient Health Questionnaire (PHQ-9) prediction. The x-axis represents pre-defined predictors and the y-axis represents the percentage increase of mean absolute error (MAE). Each feature was shuffled 100 times.

Table S10. Permutation feature importance values of the multi-layer perceptron (MLP) base-learner for Patient Health Questionnaire (PHQ-9) prediction. MAE: mean absolute error.

| **Predictor** | **Percentage increase of MAE** |
| --- | --- |
| sex1 | 0.44 |
| first_ep | 0.01 |
| flx_past | 0.08 |
| SSRI_past | <0.01 |
| AD_past | 0.53 |
| bmi | 0.30 |
| smoker | 0.74 |
| PHQ9_base | 20.11 |
| past_psychotherapy | -0.02 |
| past_referral | -0.01 |
| past_maltreatment | 0.01 |
| chd | <0.01 |
| stroke | 0.03 |
| diabetes | 0.03 |
| epilepsy | -0.02 |
| hypothyroidism | -0.01 |
| arthritis | 0.02 |
| anxiety | 0.03 |
| migraine | 0.01 |
| antihypertensive | 0.01 |
| aspirin | -0.01 |
| statins | 0.06 |
| anticoagulants | <0.01 |
| NSAIDs | 0.06 |
| anticonvulsants | <0.01 |
| hypnotics | 0.02 |
| bisphosphonates | 0.02 |
| contraceptives | 0.04 |
| age_current | 0.34 |
| ethrisk_PA | 0.36 |
| town_quintile | 0.75 |

# Feature Importance Analysis of the MLP Base-Learner for Dropout Prediction (Figure S8, Table S11)


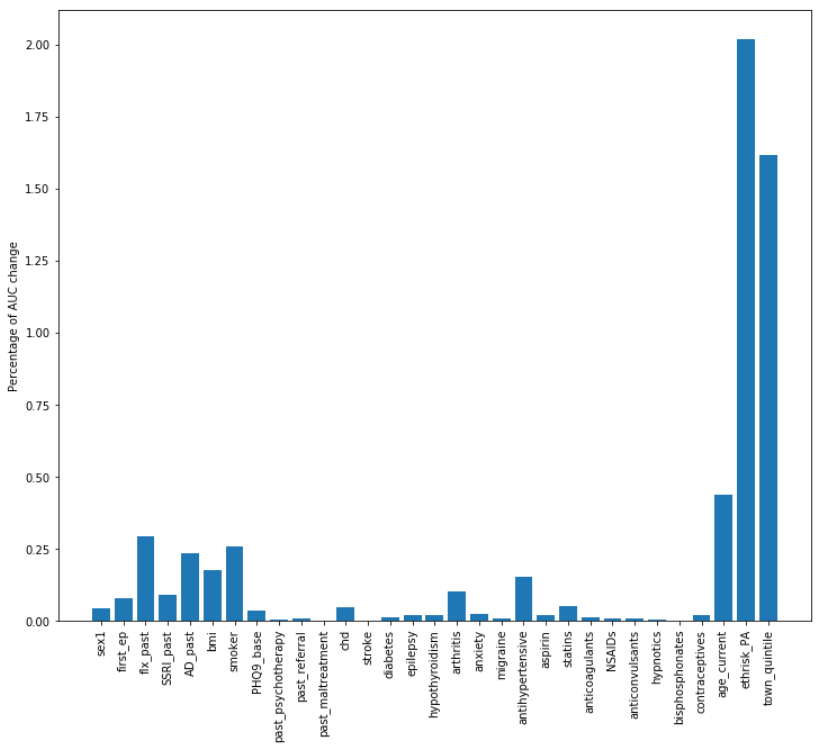


Figure S8. Permutation feature importance of the multi-layer perceptron (MLP) base-learner for all-cause treatment dropout prediction. The x-axis represents pre-defined predictors and the y-axis represents the percentage decrease of area under the receiver operating characteristic curve (AUC). Each feature was shuffled 100 times.

Table S11. Permutation feature importance values of the multi-layer perceptron (MLP) base-learner for all-cause treatment dropout prediction. AUC: area under the receiver operating characteristic curve.

| **Predictor** | **Percentage decrease of AUC** |
| --- | --- |
| sex1 | 0.05 |
| first_ep | 0.08 |
| flx_past | 0.29 |
| SSRI_past | 0.09 |
| AD_past | 0.24 |
| bmi | 0.18 |
| smoker | 0.26 |
| PHQ9_base | 0.04 |
| past_psychotherapy | <0.01 |
| past_referral | 0.01 |
| past_maltreatment | <0.01 |
| chd | 0.05 |
| stroke | <0.01 |
| diabetes | 0.01 |
| epilepsy | 0.02 |
| hypothyroidism | 0.02 |
| arthritis | 0.10 |
| anxiety | 0.04 |
| migraine | 0.01 |
| antihypertensive | 0.15 |
| aspirin | 0.02 |
| statins | 0.05 |
| anticoagulants | 0.01 |
| NSAIDs | 0.01 |
| anticonvulsants | 0.01 |
| hypnotics | <0.01 |
| bisphosphonates | <0.01 |
| contraceptives | 0.02 |
| age_current | 0.44 |
| ethrisk_PA | 2.02 |
| town_quintile | 1.62 |

# Feature Importance Analysis of the Linear Regression Meta-Learner for PHQ-9 Prediction (Figure S9)


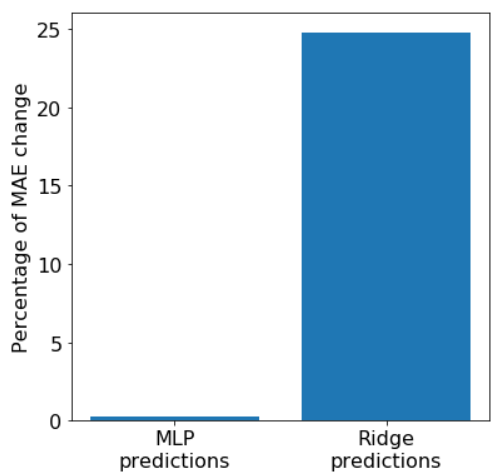


Figure S9. Permutation feature importance of the linear regression meta-learner for Patient Health Questionnaire (PHQ-9) prediction, evaluated on the meta-learner using base-learner predictions only as input. The x-axis represents pre-defined predictors and the y-axis represents the percentage increase of mean absolute error (MAE). Each feature was shuffled 100 times.

# Feature Importance Analysis of the Logistic Regression Meta-Learner for Dropout Prediction (Figure S10)


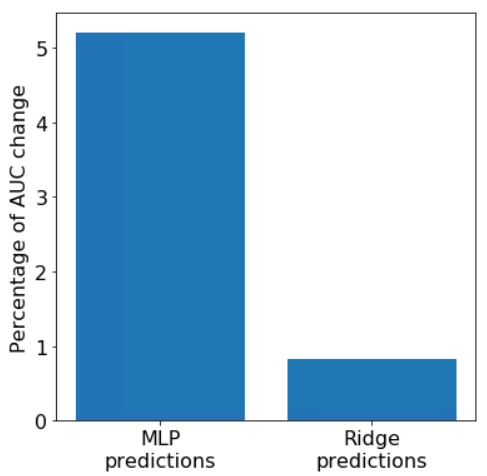


Figure S10. Permutation feature importance of the logistic regression meta-learner for all-cause treatment dropout prediction, evaluated on the meta-learner using base-learner predicted probabilities only as input. The x-axis represents pre-defined predictors and the y-axis represents the percentage decrease of area under the receiver operating characteristic curve (AUC). Each feature was shuffled 100 times.

# Feature Importance Analysis of the Ridge Regression Base-Learner for PHQ-9 Prediction (Figure S11, Table S12)


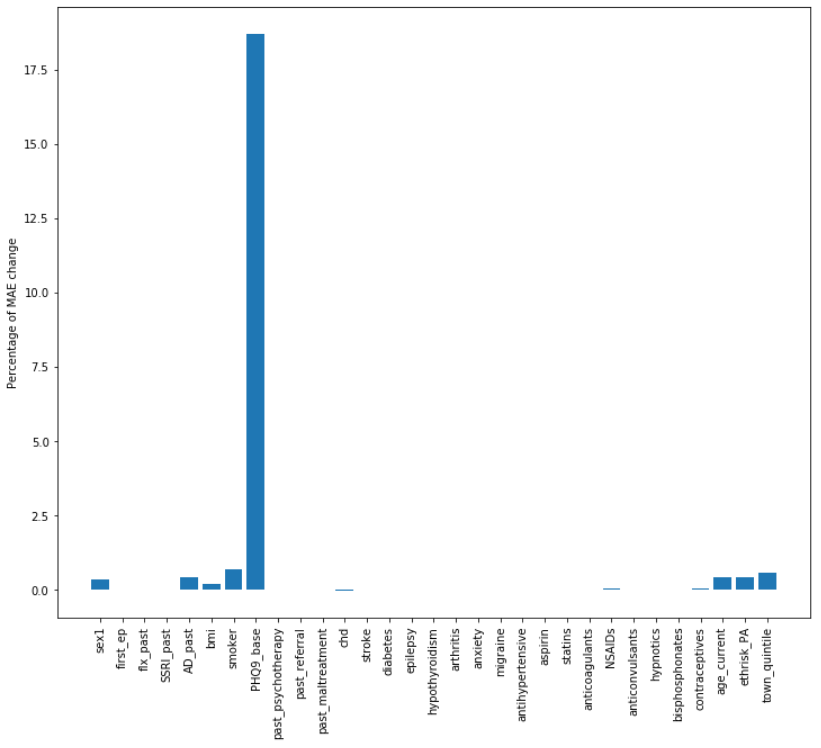


Figure S11. Permutation feature importance of the ridge regression base-learner for Patient Health Questionnaire (PHQ-9) prediction. The x-axis represents pre-defined predictors and the y-axis represents the percentage increase of mean absolute error (MAE). Each feature was shuffled 100 times.

Table S12. Permutation feature importance values of the ridge regression base-learner for Patient Health Questionnaire (PHQ-9) prediction. MAE: mean absolute error.

| **Predictor** | **Percentage increase of MAE** |
| --- | --- |
| sex1 | 0.34 |
| first_ep | 0.01 |
| flx_past | 0.02 |
| SSRI_past | 0.02 |
| AD_past | 0.41 |
| bmi | 0.21 |
| smoker | 0.69 |
| PHQ9_base | 18.68 |
| past_psychotherapy | <0.01 |
| past_referral | -0.01 |
| past_maltreatment | <0.01 |
| chd | -0.01 |
| stroke | <0.01 |
| diabetes | <0.01 |
| epilepsy | -0.01 |
| hypothyroidism | <0.01 |
| arthritis | 0.02 |
| anxiety | <0.01 |
| migraine | -0.01 |
| antihypertensive | <0.01 |
| aspirin | <0.01 |
| statins | 0.02 |
| anticoagulants | <0.01 |
| NSAIDs | 0.04 |
| anticonvulsants | 0.02 |
| hypnotics | <0.01 |
| bisphosphonates | <0.01 |
| contraceptives | 0.05 |
| age_current | 0.42 |
| ethrisk_PA | 0.42 |
| town_quintile | 0.57 |

# Feature Importance Analysis of the Ridge Regression Base-Learner for Dropout Prediction (Figure S12, Table S13)


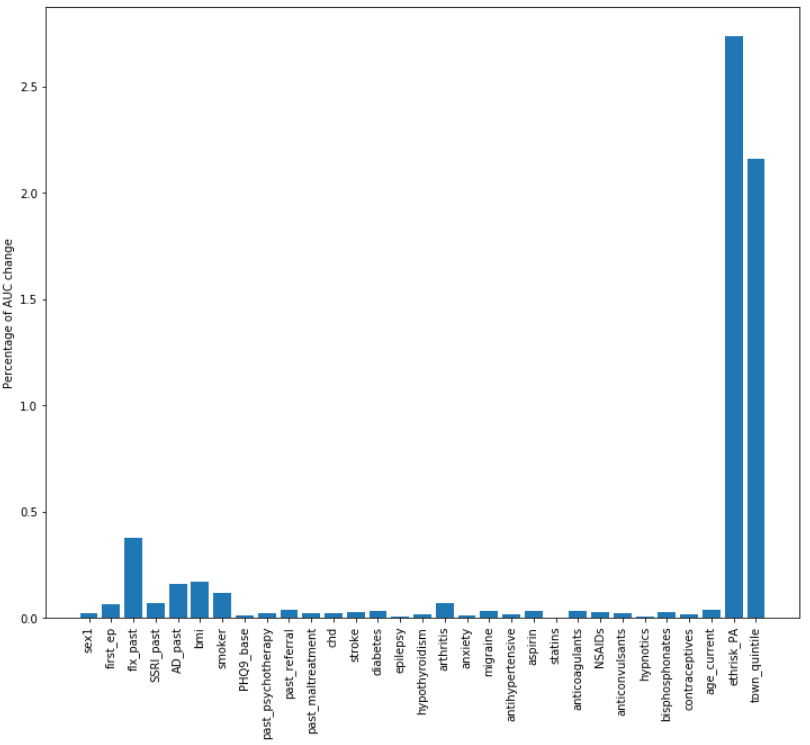


Figure S12. Permutation feature importance of the ridge regression base-learner for all-cause treatment dropout prediction. The x-axis represents pre-defined predictors and the y-axis represents the percentage decrease of area under the receiver operating characteristic curve (AUC). Each feature was shuffled 100 times.

Table S13. Permutation feature importance values of the ridge regression base-learner for all-cause treatment dropout prediction. AUC: area under the receiver operating characteristic curve.

| **Predictor** | **Percentage decrease of AUC** |
| --- | --- |
| sex1 | 0.02 |
| first_ep | 0.07 |
| flx_past | 0.38 |
| SSRI_past | 0.07 |
| AD_past | 0.16 |
| bmi | 0.17 |
| smoker | 0.12 |
| PHQ9_base | <0.01 |
| past_psychotherapy | 0.02 |
| past_referral | 0.04 |
| past_maltreatment | 0.02 |
| chd | 0.02 |
| stroke | 0.03 |
| diabetes | 0.03 |
| epilepsy | 0.01 |
| hypothyroidism | 0.02 |
| arthritis | 0.07 |
| anxiety | 0.01 |
| migraine | 0.03 |
| antihypertensive | 0.02 |
| aspirin | 0.03 |
| statins | <0.01 |
| anticoagulants | 0.03 |
| NSAIDs | 0.03 |
| anticonvulsants | 0.02 |
| hypnotics | 0.01 |
| bisphosphonates | 0.03 |
| contraceptives | 0.02 |
| age_current | 0.04 |
| ethrisk_PA | 2.74 |
| town_quintile | 2.16 |

# Evaluation of Data Pre-Processing Techniques (Table S14)

Table S14. Evaluation of data pre-processing techniques using the ridge regression base-learner for Patient Health Questionnaire (PHQ-9) prediction. MAE: mean absolute error. CI: confidence interval, calculated by the interval from the 2.5th to the 97.5th percentile.

|  | PHQ-9 score  MAE [95% CI] |
| --- | --- |
| No data pre-precessing | 4.638 [4.572, 4.709] |
| Data standardization | 4.638 [4.572, 4.709] |
| Data normalization | 4.638 [4.572, 4.709] |

# Reusing Base-Learner Predictors as Additional Predictors for the Meta-Learners (Table S15)

Table S15. Evaluation on post treatment Patient Health Questionnaire (PHQ-9) and all-cause treatment dropout predictions made by the meta-learners with patient baseline information reused as additional predictors of the meta-learners. MLP: multi-layer perceptron. MAE: mean absolute error. CI: confidence interval. AUC: area under the receiver operating characteristic curve.

|  | MAE [95% CI] | AUC [95% CI] |
| --- | --- | --- |
| Meta-learner linear/logistic regression | 4.545 [4.487, 4.612] | 0.603 [0.600, 0.606] |
| Meta-learner MLP | 4.517 [4.453, 4.487] | 0.606 [0.602, 0.609] |

# References

1. De Crescenzo F, Garriga C, Tomlinson A, Coupland C, Efthimiou O, Fazel S, Hippisley-Cox J, Cipriani A: Real-world effect of antidepressants for depressive disorder in primary care: protocol of a population-based cohort study. *Evidence-Based Mental Health* 2020, 23(3):122-126.

2. Coupland C, Dhiman P, Morriss R, Arthur A, Barton G, Hippisley-Cox J: Antidepressant use and risk of adverse outcomes in older people: population based cohort study. *BMJ* 2011, 343.

3. Coupland C, Hill T, Morriss R, Moore M, Arthur A, Hippisley-Cox J: Antidepressant use and risk of adverse outcomes in people aged 20–64 years: cohort study using a primary care database. *BMC Medicine* 2018, 16(1):1-24.

4. Malhi G, Mann J: Depression. *Lancet* 2018, 10161(392):2299–2312.

5. Steinert C, Hofmann M, Kruse J, Leichsenring F: The prospective long-term course of adult depression in general practice and the community. A systematic literature review. *Journal of Affective Disorders* 2014, 152:65-75.

6. Hegel MT, Oxman TE, Hull JG, Swain K, Swick H: Watchful waiting for minor depression in primary care: remission rates and predictors of improvement. *General Hospital Psychiatry* 2006, 28(3):205-212.

7. Moore M, Byng R, Stuart B, Harris T, Kendrick T: ‘Watchful waiting’ or ‘active monitoring’ in depression management in primary care: Exploring the recalled content of general practitioner consultations. *Journal of Affective Disorders* 2013, 145(1):120-125.

8. Iglesias-González M, Aznar-Lou I, Gil-Girbau M, Moreno-Peral P, Peñarrubia-María MT, Rubio-Valera M, Serrano-Blanco A: Comparing watchful waiting with antidepressants for the management of subclinical depression symptoms to mild–moderate depression in primary care: a systematic review. *Family Practice* 2017, 34(6):639-648.

9. Barley EA, Murray J, Walters P, Tylee A: Managing depression in primary care: a meta-synthesis of qualitative and quantitative research from the UK to identify barriers and facilitators. *BMC Family Practice* 2011, 12(1):1-11.

10. Kendrick T, King F, Albertella L, Smith PW: GP treatment decisions for patients with depression: an observational study. *British Journal of General Practice* 2005, 55(513):280-286.

11. Wiles N, Taylor A, Turner N, Barnes M, Campbell J, Lewis G, Morrison J, Peters TJ, Thomas L, Turner K: Management of treatment-resistant depression in primary care: a mixed-methods study. *British Journal of General Practice* 2018, 68(675):e673-e681.

12. Džeroski S, Ženko B: Is combining classifiers with stacking better than selecting the best one? *Machine Learning* 2004, 54(3):255-273.

13. Boehmke B, Greenwell BM: Hands-on machine learning with R: CRC Press; 2019.
